# Floral diversity and conservation status of vascular plants in arid mountainous areas

**DOI:** 10.1186/s12862-024-02342-y

**Published:** 2025-01-07

**Authors:** Sara Hassanen, Elsayeda Gamal Eldin, Wafaa Kamel, Mohamed Saad Zaghloul, Yasmin M. Hassan

**Affiliations:** Botany & Microbiology Department, Faculty of Science, Suez Canal University, Ismailia, Egypt

**Keywords:** Wild flora, Climate change, Anthropogenic interference, Conservation, Biodiversity

## Abstract

**Background:**

The destructive human activities, encroachment of natural habitats, and hyperarid climate threaten the wild flora of the unprotected mountainous areas facing the Gulf of Suez, Egypt. So, this study aims to revise and give an updated systematic status of the flowering plants growing there to conserve and utilize valuable biodiversity.

**Results:**

This study showed the presence of 136 species, including 7 sub-species of vascular plants, 12 species of monocots, and 124 species dicots belonged to 98 genera and 37 families. The most species-rich families were Asteraceae (22 species) and Amaranthaceae (19 species). Therophytes and Chamaephytes were the most dominant life- forms in the study area, representing 38.2%. They were followed by Phanerophytes, Hemicryptophytes, and Cryptophytes, which represented 11%, 8.8%, and 2.9%, respectively. Five plant assemblages were identified by TWINSAPN classification namely, *Zygophyllum coccineum* –*Haloxylon salicornicum* assemblage, *Zilla spinosa -Zygophyllum coccineum* assemblage, *Zygophyllum coccineum-Tamarix nilotica* assemblage, *Tamarix nilotica – Phargmites australis* assemblage and *Tamarix nilotica–Chenopodium murale* assemblage. Several invasive species were recorded in some wadis. However, their presence is unusual to the floristic composition of the wadis in general and acts as an alarm to protect the native species from anthropogenic interference. Moisture content, organic matter, electrical conductivity, pH, cations, anions, and total carbonate were identified as the significant factors controlling distribution of plant clusters by detrended correspondence analysis. This study recorded *Tribulus mollis* as a new addition to Egypt’s flora of Eastern desert.

**Conclusion:**

The comparative analysis of the present and past floral studies in the study area reveals a significant change in the plant community composition. This shift is likely attributed to the adverse impacts of climate change and anthropogenic activities. Thus, this area has to be safeguarded with practical strategies that aid in preserving the significant uncommon flora.

**Supplementary Information:**

The online version contains supplementary material available at 10.1186/s12862-024-02342-y.

## Introduction

The wild flora of the mountainous area in Egypt facing the Gulf of Suez is under severe threat due to various factors, including quarries, the construction of the Al-Galala-Wadi Hagul-Zafarana new road, cement factories, tourism development, and climate change. These factors have a destructive effect on the natural flora, altering the distribution of plants and leading to the extinction of some species in the study area [1–4].

Quarries and the construction of the new road have had a significant negative impact on flora and biodiversity. The use of heavy machinery and explosives has led to air pollution, dislocation and interruption of water to fertile soil, habitat destruction, and damage to flora [1]. Cement factories also contribute to the problem by producing dust and gases that contaminate the soil and negatively impact plant physiological processes, reducing plant length, leaves, and cover, and sometimes even resulting in extinction [2].

The development of the tourism industry along the Red Sea coastlines has also endangered large areas of the desert and numerous plant populations. Over 15% of the Red Sea’s coastline zone has been taken over by hotels and tourist settlements in the past ten years [3]. Some of these tourist villages and summer resorts were also established along Suez and Ain-Shokna highways till Zafarana, negatively affecting the growth and frequency of wild plants.

Climate change, characterized by high temperatures and a lack of rainfall in the study area, is another significant factor determining the presence, development, growth, distribution, and densities of plants. It restricts the availability of vital plant nutrients and crop growth and adversely affects ecological processes. Additionally, the increased soil salinization rate accompanying aridity further inhibits flora growth [4].

Despite the importance of the study region, it remains poorly investigated, and there has never been a recent comprehensive study on the flora or vegetation of the mountainous areas facing the Gulf of Suez. However, some ecological studies have been conducted on limited sites within the study area, such as Cairo-Suez Road, Ain Shokna, Wadi Hagul, and Gebel Ataqa [5–13].

Furthermore, much work must be done to improve the Egyptian flora, including updating the names of families and taxa, revising the geographic distribution of many species, and thoroughly exploring the flora of some region.

As part of a conservation approach, this study aims to determine the current floristic composition of the study region and demonstrate the extent to which it is impacted by human activity and climate change.

## Materials and methods

### Study area

**Fig. 1:**
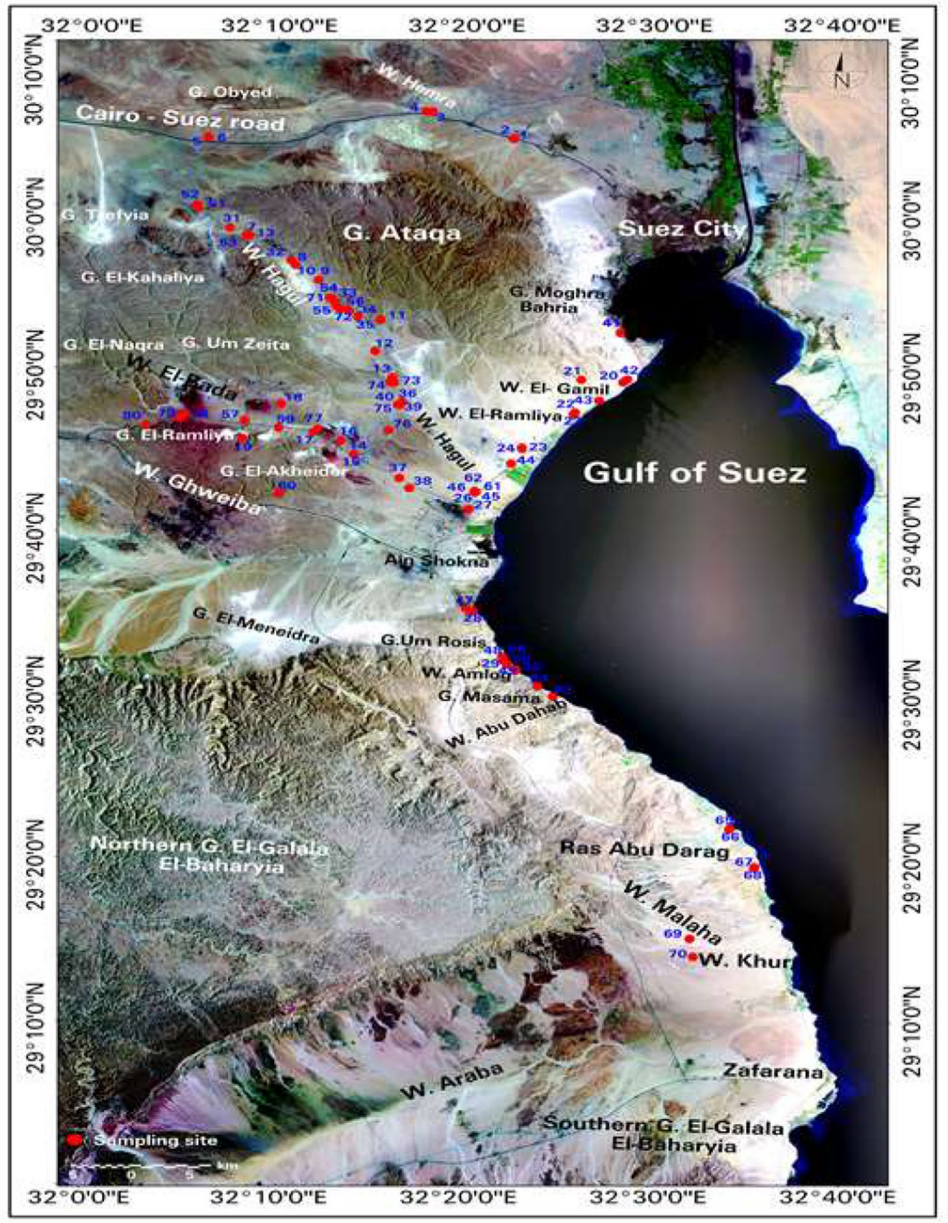
The location of the collection sites of the study area

The study area is the mountainous areas facing the North-East section of the Gulf of Suez. It is located at 30° 0’ 28.90” N, 32° 17’ 23.37” E, about 8,571.49 km^2^, extending from Cairo-Suez Road to Zafarana. It is represented by 80 collection sites including eleven wadis: Wadi Hemra, Wadi Hagul, Wadi El- Bada, Wadi Ghweiba, Wadi El-Gamil, Wadi El-Ramliya, Wadi Amlog, Wadi Malaha, Wadi Khurri, Ras Abu Darag, and Wadi Abu Dahab. In addition, it includes several mountains: Gebel Ataqa, Gebel Um Zeita, Gebel El-Ramylia, Gebel Um Rosis, Gebel El-Akheider, Gebel Masama, and Gebel Moghra Bahria (Fig. 1).

The Eastern Desert is characterized by various geomorphologic units, with structural plateaus and ridges supported by carbonate rocks. The southeastern half is primarily affected by sandstone-based structural plains, while elevated beaches and lagoonal mud cover the coastal lowlands. The basement ridge, which rises 1000 m above sea level, is the primary watershed area [14]. The desert surrounding the Red Sea is primarily mountainous, with coastal mountains on the western side. A gently sloping plain stretches between hills and coastline, with sand covering the wide coastal plain. Wadis, drainage systems, meander east, empties into the Red Sea and Gulf of Suez and drain their water [7].

The study area has a subtropical desert/low-latitude, arid, hot climate, with monthly average temperatures varying between summer and winter. The highest average temperature is 35.3 °C in summer, while the lowest is 15.3 °C in winter. Precipitation is highest in winter, with 5 mm average, while spring and summer have the lowest according to the Köppen-Geiger classification (BWh)and the Holdridge life zones system of bioclimatic classification [15].

The Eastern Desert Mountains are drained by numerous wadis, with a catchment area of around 500 km^2^. The global regulated potential is a few hundred thousand m^3^ per year per wadi, with exploitation potential not exceeding a few hundred m^3^/day. The basement complex, composed of crystalline rocks, has abundant water points, but high rainfall conditions limit water retention. Wadis are primarily drained from deeper alluvial pockets or rocky shelves [14].

This study is based on fresh specimens from their natural habitats, including the 2022 to 2024 representing 80 collection sites. The belt transect method was used in sampling of the collected plant specimens within the study area. The transects were established to covered the entire length of the wadi. Along each transect, we recorded the presence or absence of all plant species encountered. All specimens are conserved and preserved in the Suez Canal University Herbarium (SCU-I). (3–7) specimens vouchered each taxon observed in the field. The studied specimens were identified by Boulos [16–19] **&** [20]. The specimens were also compared with herbarium sheets kept in the Suez Canal University Herbarium (SCU-I). In addition to using the plant flora of different neighboring countries were also used to achieve an accurate identification [21] **&** [22]. The recent valid names of the recorded taxa were revised and verified with international reference databases [23]. The geographical distribution of the recorded species in Egypt and the world [24].

Three soil samples were taken from each collection site to undergo some vegetation analysis. The soil samples were collected from a depth of 0–30 cm using a shovel. The samples were placed on sheets of paper to be air-dried for one week and mixed to make one composite soil sample. The composite soil samples were passed through a 2 mm sieve to get rid of gravels and debris. Physical analysis performed by using the pipette method [25]. Organic matter and Organic carbon percentage were determined by using Walkley and Black method [26]. Soil samples were subjected to chemical soil analysis. The saturated soil paste extract’s electrical conductivity (EC) was measured in dsm^− 1^ using the meter model Jenway 3310 [27]. Soil pH was measured in a 1:2.5 soil-to-water suspension using a bench-type Beckman glass electrode pH meter [28]. Calcium and magnesium were extracted from the saturated soil using volumetric titration with ethylenediaminetetraacetic acid (EDTA) and measured in mEq/L. Sodium and potassium were measured using a flame photometer. Bicarbonate was titrated with sulfuric acid and measured in mEq/L [28], while chloride was measured in mEq/L using silver nitrate. The total calcium carbonate percentage was calculated using Collin’s calcimeter [29].

The phytosociological data set, consisting of 80 sites and 136 species, was classified using the TWINSPAN (Two-way Indicator Species Analysis) technique in the PC-ORD computer program [30], version 4.01 for Windows. This multivariate analysis technique arranges multivariate data in an ordered two-way table by classification of individuals and attributes. To interpret the species/environment relationships, ordination was carried out using DCA technique in PC-ORD computer program [30], version 4.01 for Windows Detrended Correspondence Analysis is a multivariate method used for sorting species and samples along environmental gradients.

**Fig. 2:**
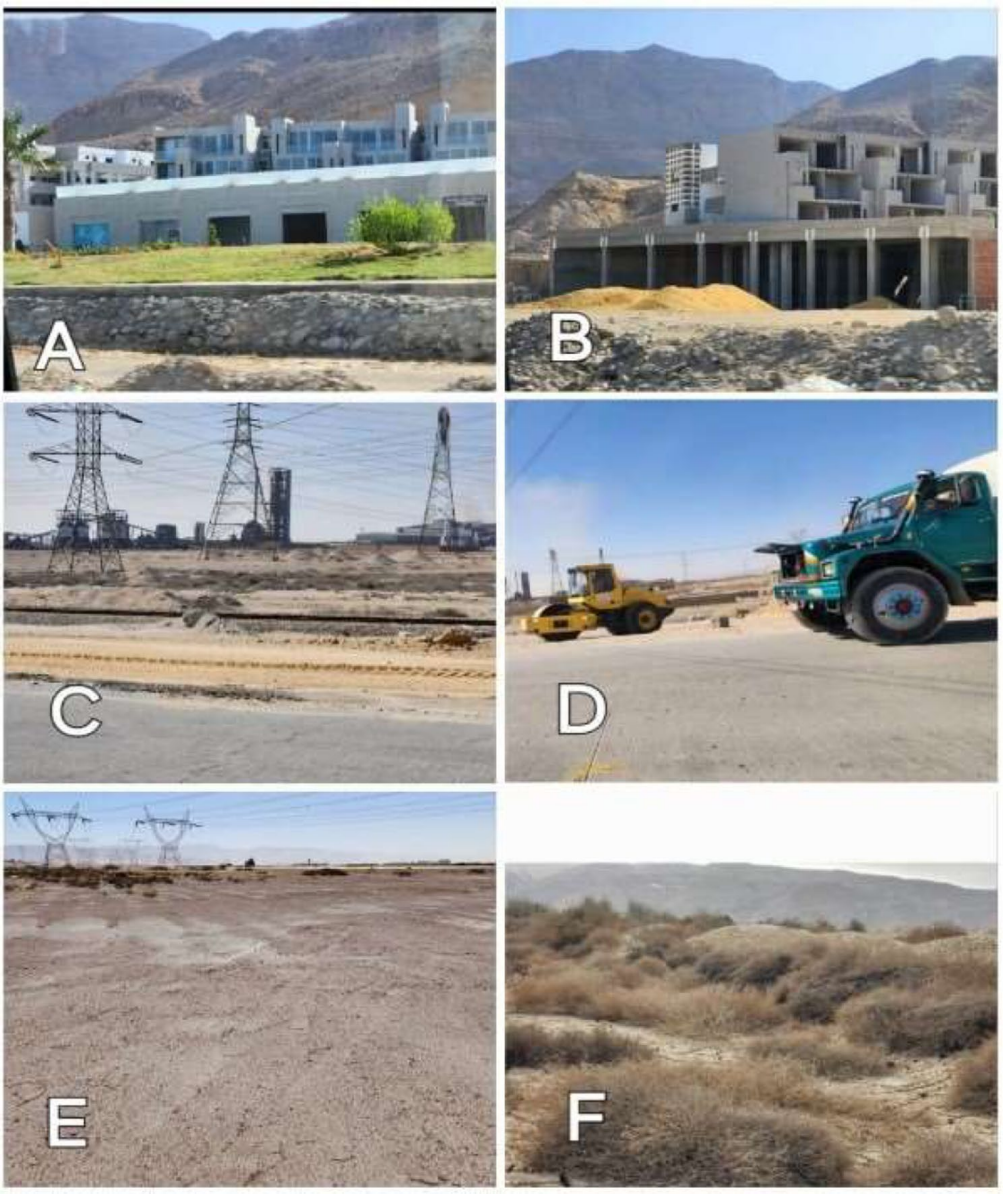
Various types of anthropogenic interference that lead to habitat loss for the natural wild flora. (**A**&**B**): Summer resorts Infront of mountains. (**C**&**D**): Quarries and Building of Al-Galala–Wadi Hagul–Zafarana new road (Distance between site 46^th^ and 47^th^). (**E**): Cement factories alter the soil’s composition, making it harder and not conducive to plant life. (Site 27^th^). (**F**): The hyper arid climate effect on plants

It must be stated that the flora of the study area suffers from the harmful side effects of quarries and cement factories. Some habitats of the wild flora in the study area were destroyed completely due to the work of heavy machinery. Subsequently, all the wild plants were completely eroded. This was obvious in the area between the 46th and 47th sites, represented by 13.222 km^2^. (Fig. 2C and D)

Great evidence of the disastrous effects of cement manufacturers may be seen at site 27th. The plants were unable to survive, and it was very arid as the soil became extremely hard, like cement. So, it was tough to take a soil sample from this site (Fig. 2E).

## Results

**Fig. 3:**
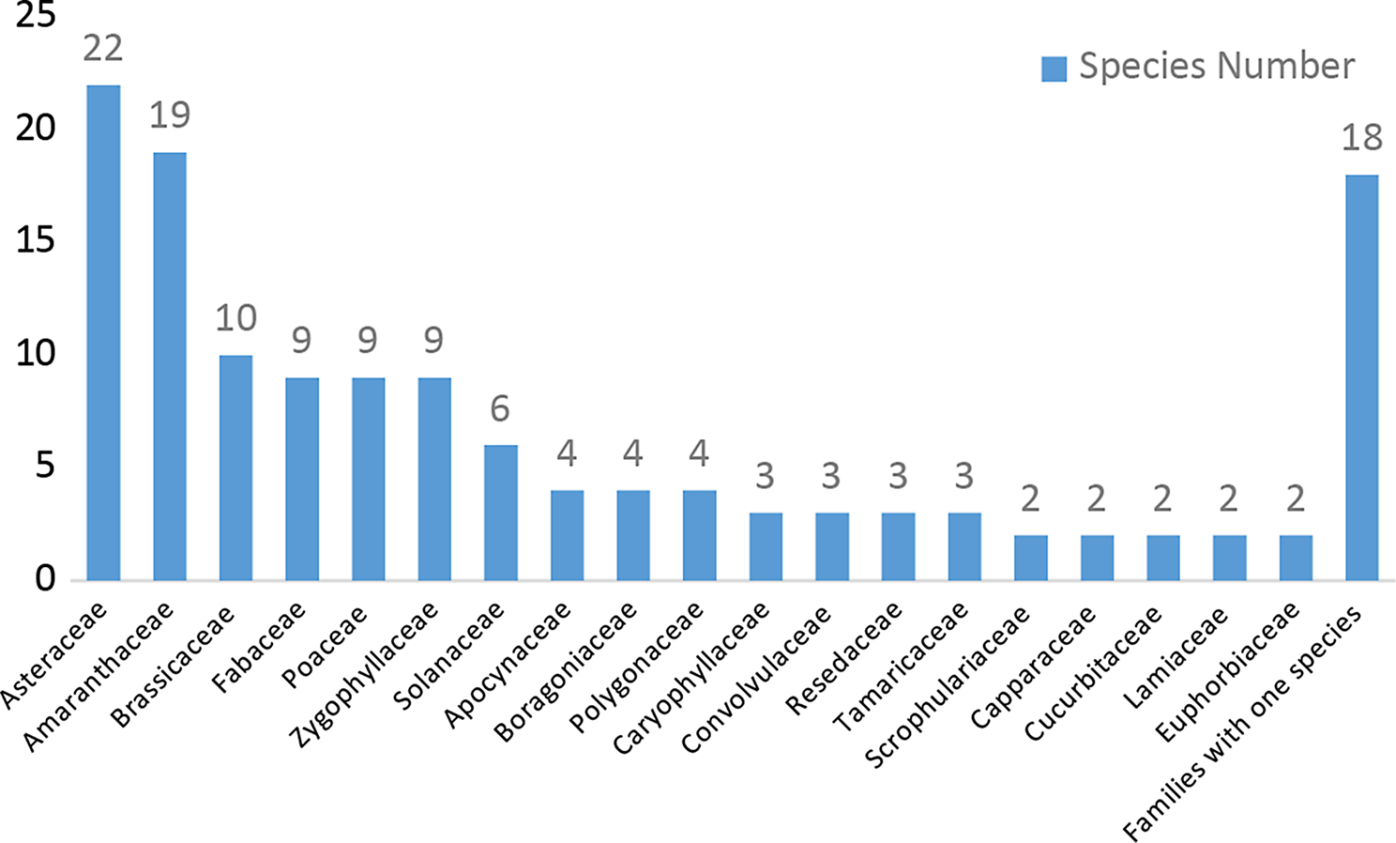
Most species-rich families with the number of species

In the study area, the species composition revealed 136 species, including 7 sub-species; these species were divided into 124 dicot and 12 monocot species, belonging to 37 families and 98 genera of vascular plants. They included three trees, 12 shrubs, 84 perennials, 2 biennials, and 50 annuals. The most species- rich families were Asteraceae (22 species), Amaranthaceae (19 species) and Brassicaceae (10 species). They represented 16.1%, 13.9%, and 7.3% of total collected samples, followed by Fabaceae, Poaceae, and Zygophyllaceae (9 species). The fewer species families represented by Boragoniaceae (4 species), Caryophyllaceae (3 species), and Capparaceae (2 species). Finally, twenty-one families were monogeneric: Asphodelaceae, Neuradaceae, Ephedraceae and Plumbaginaceae. (Table 1; Fig. 3).

The size of the families is typically small because just two families have more than ten species: Asteraceae and Amaranthaceae. The well-represented genera were *Zygophyllum* (7 species), *Launaea* (4 species), *Chenopodium*,* Bassia*,* Tamarix*,* Heliotropium*,* Rumex*,* Hyoscyamus*, and *Haloxylon* (3 species), *Tribulus*,* Cleome* and *Artemisia* (2 species). But, 18 genera were each represented by only one species e.g., *Anastatica*,* Cucumis*,* Nitraria*, and *Salvia.* The perennials represented most of the collected samples by 61.7%, among them *Astragalus dactylocarpus*,* Retama raetam*,* Lavandula coronopifolia*,* Panicum turgidum*,* Haplophyllum tuberculatum*,* Kickxia aegyptiaca* and *Ochradenus baccatus.* Shrubs represented 8.8% of collected samples, including *Lycium shawii*,* Nitraria retusa* and *Tamarix nilotica*, while the trees were represented by three taxa: *Phoenix dactylifera*,* Vachellia tortilis* subsp. *raddiana*, and *Vachellia tortilis* subsp. *tortilis.* On the other hand, the annuals represented 36.76% of the recorded species among them: *Caylusea hexagyna*,* Scrophularia deserti*,* Plantago ovata* and *Monsonia nivea.* In the study area, some species show high occurrence e.g. *Zygophyllum coccineum* was recorded in the 61 sites and represented 76.25% showing the highest occurrence among the recorded species, followed by *Zilla spinosa* (50 sites, 62.5%), *Ochradenus baccatus* (46 sites, 57.5%), *Tamarix nilotica* (38 sites, 47.5%) and *Leptadenia pyrotechnica* (16 sites, 20%) On the other hand, *Neurada procumbens*, *Blepharis edulis*,* Asphodelus tenuifolius* and *Halopeplis perfoliata* (one site, 1.25%) showing the lowest occurance.

**Fig. 4:**
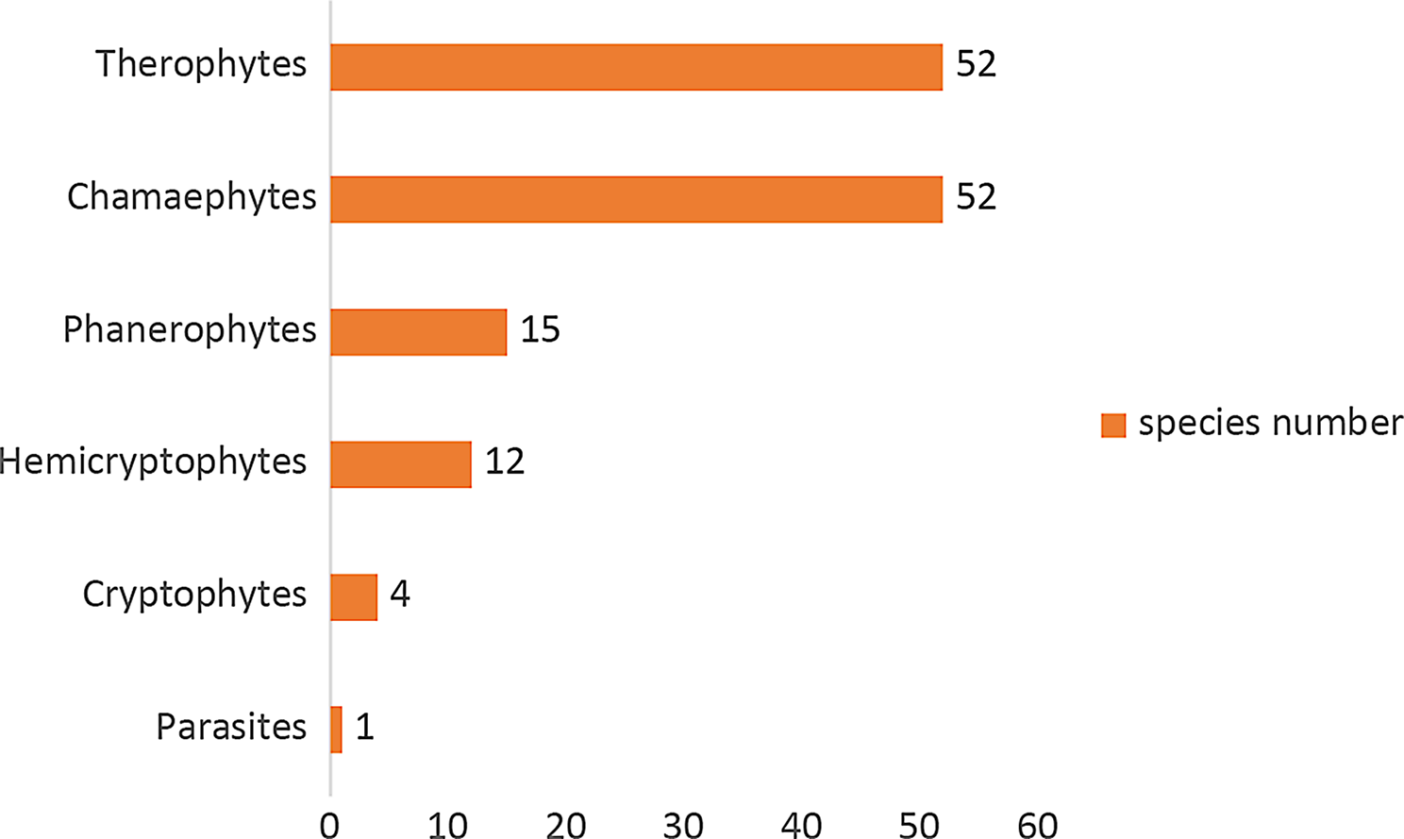
The number of the different life forms in the study area

The total number of species recorded in the eleven wadis of the study area is shown (Table 1; Fig. 4). The highest number of species were recorded in Wadi Hagul (88 species, 64.7%) followed by Wadi El-Bada (41 species, 30.1%), then Wadi El-Ramliya (33 species, 24.2%). Some species were recorded in all wadis showing great dominance e.g., *Zygophyllum coccineum*,* Zilla spinosa*, and *Tamarix nilotica*. But some species were restricted to only one wadi e.g., *Gymnocarpos decander*, *Neurada procumbens*, *Salvia aegyptiaca*,* Tribulus mollis*, *Asphodelus tenuifolius* and *Plantago ovata*, which were restricted to Wadi Hagul representing 14.7%. Indicating that it has the largest number of different species. While *Chenopodiun murale*, *Lysimachia arvensis*, *Solanum lycopersicum* are restricted to Ras Abu Darag, representing 8.8%. Moreover, *Zygophyllum bruguieri*, *Atriplex humilis*, and *Caylusea hexagyna* were restricted to Wadi El-Bada, representing 2.9%.

**Fig. 5:**
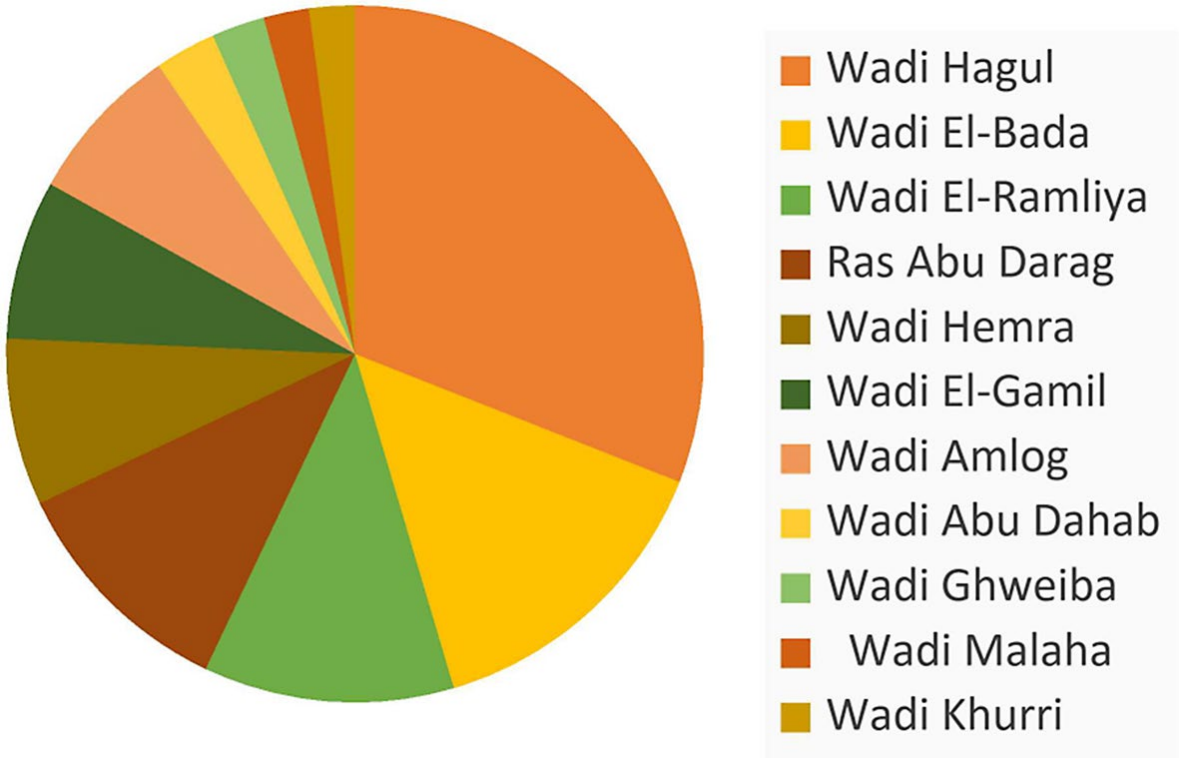
The species distribution among eleven wadis

The life forms spectrum of the study area showed some variations. Therophytes and Chamaephytes were the highest life forms sharing the same percentage of 38.2%. Followed by Phenerophytes, which represented 11%, followed by hemicryptophytes, and cryptophytes, which represented 8.8% and 2.9%. At last, the parasites represented 0.7%. The distribution of the different life forms in the two sections of the study area was shown (Table 1; Fig. 5).

**Table 1:** List of recorded species in the study area, life forms, habitats and geographical distribution of the recorded species

Family	Species	Duration	Life form	Wadi Hagul	Wadi El-Bada	Wadi Hemra	Wadi El-Gamil	Wadi El-Ramliya	Wadi Amlog Wadi Amlog Wadi Amlog Wadi Amlog	Wadi Ghweiba	Wadi Abu Dahab	Ras Abu Darag	Wadi Malaha	Wadi Khurri
Acanthaceae	*Blepharis edulis* (Forssk.) Pers.	Per	Ch	**-**	*-*	***+***	**-**	**-**	*-*	*-*	*-*	*-*	*-*	*-*
Amaranthaceae	*Aerva javanica* (Burm.f.) Juss. ex Schult. var *javanica*	Per	Ch	*-*	***+***	*-*	*-*	*-*	*-*	*-*	*-*	*-*	*-*	*-*
	*Amaranthus viridis* L.	Ann	Th	*-*	*-*	*-*	*-*	*-*	*-*	*-*	*-*	***+***	*-*	*-*
	*Anabasis articulata* (Forssk.) Moq.	Per	Ch	***+***	*-*	*-*	*-*	*-*	*-*	*-*	*-*	*-*	*-*	*-*
	*A. setifera* Moq.	Per	Ch	***+***	***+***	***+***	***+***	*-*	*-*	***+***	*-*	*-*	*-*	*-*
	*Arthrocaulon macrostachyum* (Moric.) Piirainen & G. Kadereit	Per	Ch	*-*	*-*	*-*	*-*	*-*	***+***	*-*	*-*	*-*	*-*	*-*
	*Atriplex humilis* F.Muell.	Per	Ch	*-*	***+***	*-*	*-*	*-*	*-*	*-*	*-*	*-*	*-*	*-*
	*Bassia eriophora* (Schrad.) Asch.	Ann	Th	***+***	*-*	*-*	*-*	*-*	*-*	*-*	*-*	***+***	*-*	*-*
	*B. indica* (Wight) A.J.Scott	Ann	Th	***+***	***+***	***+***	*-*	***+***	***+***	*-*	*-*	***+***	*-*	*-*
	*B. muricata* (L.) Asch.	Ann	Th	*-*	***+***	*-*	***+***	*-*	*-*	*-*	*-*	*-*	*-*	*-*
	*Caroxylon imbricatum* (Forssk.) Moq.	Per	Ph	***+***	*-*	*-*	***+***	*-*	*-*	*-*	*-*	*-*	*-*	*-*
	*C. inerme* (Forssk.) Akhani & Roalson	Ann	Th	*-*	*-*	*-*	*-*	*-*	***+***	*-*	*-*	*-*	*-*	*-*
	*Chenopodium album* L.	Ann	Th	*-*	*-*	*-*	*-*	*-*	*-*	*-*	*-*	***+***	*-*	*-*
	*C. ficifolium* Sm.	Ann	Th	*-*	*-*	*-*	*-*	*-*	*-*	*-*	*-*	**+**	*-*	*-*
	*C. murale* L.	Ann	Th	*-*	*-*	*-*	*-*	*-*	*-*	*-*	*-*	**+**	*-*	*-*
	*Halopeplis perfoliata* (Forssk.) Bunge ex Ung.-Sternb.	Ann	Th	*-*	*-*	*-*	*-*	*-*	*-*	*-*	*-*	*-*	***+***	*-*
	*Haloxylon persicum* Bunge	Per	Ph	***+***	*-*	*-*	*-*	*-*	*-*	*-*	*-*	*-*	*-*	***+***
	*H. salicornicum* (Moq.) Bunge ex Boiss.	Per	Ch	***+***	***+***	***+***	***+***	***+***	***+***	***+***	*-*	*-*	*-*	*-*
	*H. scoparium* pomel.	Per	Ch	*-*	***+***	***+***	*-*	*-*	*-*	***+***	*-*	*-*	*-*	*-*
	*Traganum nudatum* Delile	Per	Ch	***+***	*-*	*-*	***+***	***+***	*-*	*-*	*-*	*-*	*-*	*-*
Apiaceae	*Deverra tortuosa* (Desf.) DC.	Per	Ch	***+***	***+***	*-*	*-*	*-*	*-*	*-*	*-*	*-*	*-*	*-*
Apocynaceae	*Calotropis procera* (Aiton) W.T. Aiton.	Per	Ch	***+***	*-*	*-*	*-*	***+***	*-*	*-*	*-*	*-*	*-*	*-*
	*Cynanchum acutum* L.subsp. *acutum*	Per	H	***+***	***+***	*-*	*-*	***+***	*-*	*-*	***+***	*-*	*-*	*-*
	*Leptadenia pyrotechnica (*Forssk.) Decne.	Per	Ph	*+*	*+*	*-*	*-*	*+*	*+*	*-*	*+*	*-*	*-*	*-*
	*Pergularia tomentosa* L.	Per	Ch	*+*	*+*	*-*	*-*	*-*	*-*	*-*	*-*	*-*	*-*	*-*
Arecaceae	*Phoenix dactylifera* L.	Per	Ph	*-*	*-*	*-*	*-*	*-*	*-*	*-*	*-*	*+*	*-*	*-*
Asphodelaceae	*Asphodelus tenuifolius* Cav.	Ann	Th	*+*	*-*	*-*	*-*	*-*	*-*	*-*	*-*	*-*	*-*	*-*
Asteraceae	*Achillea fragrantissima* (Forssk.) Sch.Bip.	Per	Ch	*+*	*-*	*-*	*-*	*+*	*-*	*-*	*-*	*-*	*-*	*-*
	*Artemisia judaica* L.	Per	Ch	*+*	*-*	*-*	*-*	*-*	*-*	*-*	*-*	*-*	*-*	*-*
	*A. monosperma* Delile, Descr.	Per	Ch	*-*	*-*	*+*	*-*	*-*	*-*	*-*	*-*	*-*	*-*	*-*
	*Brocchia cinerea* (Delile) Vis.	Ann	Th	*+*	*-*	*-*	*-*	*-*	*-*	*-*	*-*	*-*	*-*	*-*
	*Centaurea aegyptiaca* L.	Bi	Th	*+*	*-*	*+*	*-*	*-*	*-*	*-*	*-*	*-*	*-*	*-*
	*C. pallescens* Delile.	Ann	Th	*+*	*-*	*-*	*-*	*-*	*+*	*-*	*-*	*-*	*-*	*-*
	*Echinops spinosus* L.	Per	H	*+*	*+*	*-*	*-*	*+*	*+*	*-*	*-*	*-*	*-*	*-*
	*Erigeron bonariensis* L.	Ann	Th	*-*	*+*	*-*	*-*	*-*	*-*	*-*	*-*	*+*	*-*	*-*
	*Ifloga spicata* (Forssk.) Sch.Bip. subsp. *spicata*	Ann	Th	*+*	*-*	*-*	*-*	*-*	*-*	*-*	*-*	*-*	*-*	*-*
	*Iphiona mucronata* (Forssk.) Asch. & Schweinf.	Per	Ch	*+*	*+*	*-*	*-*	*-*	*-*	*-*	*-*	*-*	*-*	*-*
	*Launaea mucronata* (Forssk.) Muschl subsp *mucronata*	Per	Ch	*+*	*+*	*+*	*+*	*+*	*-*	*-*	*-*	*-*	*-*	*-*
	*L. nudicaulis* (L.) Hook.f.	Per	H	*+*	*-*	*-*	*-*	*-*	*-*	*-*	*-*	*-*	*-*	*-*
	*L. procumbens* (Roxb.) Ramayya & Rajagopal	Ann	Th	*-*	*-*	*-*	*-*	*-*	*-*	*-*	*-*	*-*	*-*	*-*
	*L. spinosa* (Forsk.) Sch.Bip. ex Kuntze	Per	Ch	*+*	*+*	*-*	*-*	*-*	*-*	*-*	*-*	*-*	*-*	*-*
	*Nidorella aegyptiaca* (L.) J.C.Manning & Goldblatt.	Ann	Th	*+*	*-*	*-*	*-*	*-*	*-*	*-*	*-*	*-*	*-*	*-*
	*Pluchea dioscoridis* (L.) DC.	Per	Ph	*+*	*-*	*-*	*+*	*+*	*-*	*-*	*-*	*-*	*-*	*-*
	*Pulicaria incisa* (Lam.) DC.	Per	Ch	*+*	*-*	*-*	*-*	*-*	*-*	*-*	*+*	*-*	*-*	*-*
	*P. undulata* (L.*)* C. A. Mey.subsp. *undulata*	Per	Ch	*+*	*+*	*-*	*-*	*+*	*-*	*-*	*-*	*-*	*-*	*-*
	*Reichardia tingitana* (L.) Roth	Ann	Th	*-*	*-*	*-*	*-*	*-*	*-*	*-*	*-*	*-*	*-*	*-*
	*Senecio glaucus* L.subsp *coronopifolius* (Maire.) C. Alexander.	Ann	Th	*-*	*-*	*-*	*+*	*-*	*-*	*-*	*-*	*-*	*-*	*-*
	*Sonchus oleraceus* L.	Ann	Th	*-*	*-*	*-*	*+*	*-*	*-*	*-*	*-*	*-*	*-*	*-*
	*Urospermum picroides* (L.) F.W. Schmidt.	Ann	Th	*-*	*-*	*-*	*-*	*-*	*-*	*-*	*-*	*+*	*-*	*-*
Aizoaceae	*Aizoon canariense* L.	Ann	Th	*+*	*-*	*-*	*-*	*-*	*-*	*-*	*-*	*-*	*-*	*-*
Boragoniaceae	*Heliotropium arbainense* Fresen.	Per	Ch	*+*	*-*	*-*	*-*	*-*	*-*	*-*	*-*	*-*	*-*	*-*
	*H. bacciferum* Forssk.var *bacciferum*	Per	Ch	*+*	*-*	*+*	*-*	*-*	*-*	*-*	*-*	*-*	*-*	*-*
	*H. digynum* (Forssk.) Christens	Per	Ch	*+*	*-*	*-*	*-*	*-*	*-*	*-*	*-*	*-*	*-*	*-*
	*Trichodesma africanum* (L.) Sm.	Ann	Th	*+*	*-*	*-*	*-*	*-*	*+*	*+*	*+*	*+*	*-*	*-*
Brassicaceae	*Anastatica hierochuntica* L.	Ann	Th	*+*	*-*	*+*	*-*	*-*	*-*	*-*	*-*	*-*	*-*	*-*
	*Coincya tournefortii* (Gouan) Alcaraz, T.E.Díaz, Rivas Mart. & Sánchez-Gómez	Ann	Th	*-*	*-*	*-*	*-*	*-*	*-*	*-*	*-*	*-*	*-*	*-*
	*Diplotaxis acris* (Forssk.) Boiss.	Ann	Th	*-*	*-*	*-*	*-*	*-*	*-*	*-*	*-*	*-*	*-*	*-*
	*D. harra* (Forssk.) Boiss.	Per	H	*-*	*-*	*+*	*-*	*-*	*-*	*-*	*-*	*-*	*-*	*-*
	*Eremobium aegyptiacum* (Spreng.) Asch. var *aegyptiacum.*	Bi	Th	*-*	*+*	*+*	*-*	*-*	*-*	*-*	*-*	*-*	*-*	*-*
	*Farsetia aegyptia* Turra, Farset.	Per	Ch	*+*	*-*	*-*	*-*	*+*	*-*	*-*	*-*	*-*	*-*	*-*
	*Lepidium didymum* L.	Ann	Th	*-*	*-*	*-*	*-*	*-*	*-*	*-*	*-*	*+*	*-*	*-*
	*Matthiola longipetala* (Vent.) DC.subsp. *bicornis* (Sm.) P.W. Ball.	Ann	Th	*+*	*-*	*-*	*-*	*-*	*-*	*-*	*-*	*-*	*-*	*-*
	*Matthiola longipetala* (Vent.) DC. subsp. *livida* (Delile.) Maire, DC.	Ann	Th	*+*	*-*	*-*	*-*	*-*	*-*	*-*	*-*	*-*	*-*	*-*
	*Zilla spinosa* (L.) Prantl.	Per	Ch	*+*	*+*	*+*	*+*	*+*	*+*	*+*	*+*	*+*	*+*	*+*
Capparaceae	*Cleome amblyocarpa* Barratte & Murb.	Ann	Th	*+*	*-*	*+*	*-*	*-*	*-*	*-*	*-*	*-*	*-*	*-*
	*C. droserifolia* (Forssk.) Delile	Per	Ch	*+*	*+*	*-*	*-*	*+*	*-*	*-*	*-*	*-*	*-*	*-*
Caryophyllaceae	*Gypsophila capillaris* (Forssk.) C.Chr.	Per	H	*+*	*-*	*-*	*-*	*-*	*-*	*-*	*-*	*-*	*-*	*-*
	*Gymnocarpos decander* Forssk.	Per	Ch	*+*	*+*	*-*	*-*	*-*	*-*	*-*	*-*	*-*	*-*	*-*
	*Paronychia sinaica* Fresen.	Per	H	*+*	*-*	*-*	*-*	*-*	*-*	*-*	*-*	*-*	*-*	*-*
Convolvulaceae	*Convolvulus hystrix* Vahl.	Per	Ch	*+*	*+*	*-*	*-*	*+*	*-*	*-*	*-*	*-*	*-*	*-*
	*C. lanatus* Vahl.	Per	Ch	*+*	*-*	*-*	*-*	*+*	*+*	*-*	*-*	*-*	*-*	*-*
	*Ipomoea pes-caprae* (L.) R.Br.	Per	H	*-*	*-*	*-*	*-*	*-*	*-*	*-*	*-*	*-*	*-*	*-*
Cucurbitaceae	*Citrullus colocynthis* (L.) Schrad.	Per	H	*+*	*+*	*-*	*-*	*-*	*+*	*-*	*-*	*-*	*-*	*-*
	**Cucumis melo L.	Ann	Th	*-*	*-*	*-*	*-*	*-*	*-*	*-*	*-*	*-*	*-*	*-*
Ephedraceae	*Ephedra alata* Decne.	Per	Ch	*+*	*-*	*-*	*-*	*-*	*-*	*-*	*-*	*-*	*-*	*-*
Euphorbiaceae	*Euphorbia peplus* L.	Ann	Th	*-*	*-*	*-*	*-*	*-*	*-*	*-*	*-*	*+*	*-*	*-*
	*E. retusa* (L.) Forssk.	Per	Ch	*+*	*-*	*-*	*-*	*-*	*-*	*-*	*-*	*-*	*-*	*-*
Fabaceae	*Alhagi graecorum* Boiss.	Per	Ch	*-*	*-*	*-*	*-*	*+*	*-*	*-*	*-*	*-*	*-*	*-*
	*Astragalus sieberi* DC.	Per	Ch	*+*	*-*	*-*	*-*	*-*	*-*	*-*	*-*	*-*	*-*	*-*
	*A. spinosus* (Forssk.) Muschl.	Per	Ch	*-*	*-*	*-*	*-*	*-*	*-*	*-*	*-*	*-*	*-*	*-*
	*Crotalaria aegyptiaca* Benth.	Per	Ch	*+*	*-*	*-*	*-*	*-*	*+*	*-*	*-*	*-*	*-*	-
	*Melilotus indicus* (L.) All.	Ann	Th	-	-	-	-	-	-	-	*+*	-	-	-
	*Retama raetam* (Forssk.) Webb & Berthel.	Per	Ph	*+*	*+*	*-*	*-*	*+*	*-*	*-*	*-*	-	-	-
	*Taverniera aegyptiaca* Boiss.	Per	Ch	*+*		*-*	*-*	-	*+*	-	-	-	-	-
	*Vachellia tortilis* subsp. *raddiana* (Savi) Kyal. & Boatwr.	Per	Ph	*+*	*+*	*+*	-	-	*+*	-	-	-	-	-
	*V. tortilis* subsp. *tortilis*	Per	Ph	*+*	*+*	*+*	-	-	*+*	-	-	-	-	-
Geraniaceae	*Monsonia nivea*	Per	H	-	-	-	-		-	-	*+*	-	-	-
Juncaceae	*Juncus rigidus* Desf.	Per	C	*-*	*-*	*-*	*-*	*-*	*-*	*-*	*-*	*-*	*-*	*-*
Lamiaceae	*Lavandula coronopifolia* Poir.	Per	Ch	*+*	-	-	-	-	-	-	-	-	-	-
	*Salvia aegyptiaca* L.	Per	Ch	*+*	-	-	-	-	-	-	-	-	-	-
Malvaceae	*Malva parviflora* L.	Ann	Th	-	-	-	-	-	-	-	*+*	-	-	-
Neuradaceae	*Neurada procumbens L.*	Ann	Th	*+*	-	-	-	-	-	-	-	-	-	-
Nitrariaceae	*Nitraria retusa* (Forssk.) Asch.	Per	Ph	*+*	-	-	*+*	*+*	-	-		-	-	-
Orobanchaceae	*Cistanche tubulosa* (Schenk) Wight ex Hook.f var. *tubulosa*	Per	P	+	-	-	-	+	-	-	-	-	-	-
Plantaginaceae	*Plantago ovata* Forssk.	Per	Ch	*+*	*-*	*-*	*-*	*-*	*-*	*-*	*-*	*-*	*-*	-
Plumbaginaceae	*Limonium pruinosum* (L.) Chaz.	Ann	Th	*-*	*-*	*-*	*+*	*-*	*-*	*-*	*-*	*-*	*-*	-
Poaceae	*Cenchrus biflorus* Roxb.	Per	Ch	*-*	*-*	*-*	*-*	*-*	*-*	*-*	*-*	*+*	*-*	-
	*C. divisus* (J.F.Gmel.) Verloove, Govaerts & Buttler	Per	C	*-*	*-*	*-*	*-*	*-*	*-*	*-*	*-*	*-*	*-*	-
	*Cynodon dactylon* (L.) Pers.	Per	Ch	*-*	*-*	*-*	*-*	*-*	*-*	*-*	*-*	*+*	*-*	-
	*Diplachne fusca* (L.) P.Beauv. ex Roem. & Schult.	Per	C	*-*	*-*	*-*	*-*	*-*	*-*	*-*	*-*	*-*	*-*	-
	*Imperata cylindrica* (L.) Raeusch.	Per	H	*+*	*-*	*-*	*-*	*+*	*-*	*-*	*-*	*+*	*-*	-
	*Lasiurus scindicus* Henrard.	Per	H	*+*	*+*	*-*	*-*	*-*	*-*	*-*	*-*	*-*	*-*	-
	*Panicum turgidum* Forssk.	Per	C	*+*	*+*	*-*	*-*	*-*	*+*	*-*	*-*	*-*	*-*	-
	*Phragmites australis* (L.) (Cav.) Trin.ex. Steud.	Per	H	*+*	*+*	*-*		*+*	*+*	*-*	*-*	*-*	*+*	*+*
	*Tricholaena teneriffae* (L.f.) Link	Per	Ph	*-*	*-*	*-*	*-*	*-*	*-*	*-*	*-*	*-*	*-*	*-*
Polygonaceae	*Calligonum comosum* L’Hér	Ann	Th	*+*	*-*	*-*	*-*	*-*	*-*	*-*	*-*	*-*	*-*	-
	*Rumex cyprius* Murb.	Ann	Th	*-*	*-*	*-*	*-*	*-*	*-*	*-*	*-*	*-*	*-*	-
	*R. spinosus* L.	Ann	Th	*-*	*-*	*-*	*+*	*-*	*-*	*-*	*-*	*-*	*-*	-
	*R. vesicarius* L.	Ann	Th	*+*	*-*	*-*	*+*	*-*	*-*	*-*	*-*	*-*	*-*	-
Portulacaceae	*Portulaca oleracea* L.	Ann	Th	-	-	-	-	-	-	-	*-*	+	-	-
Primulaceae	*Lysimachia arvensis* (L.) U.Manns & Anderb.	Ann	Th	-	-	-	-	-	-	-	*-*	+	-	-
Resedaceae	*Caylusea hexagyna* (Forssk.) M.L.Green	Ann	Th	-	*+*	-	-	-	-	-	-	-	-	-
	*Ochradenus baccatus* Delile	Per	Ph	*+*	*+*	*+*	*+*	*+*	*+*	*+*	*-*	+	-	-
	*Reseda pruinosa* Delile	Ann	Th	-	-	-	-	-	-	*+*	*-*	*-*	*-*	-
Rutaceae	*Haplophyllum tuberculatum* (Forssk.) A.Juss.	Per	Ch	*+*	-	-	-	-	-	-	-	-	-	-
Scrophulariaceae	*Kickxia aegyptiaca* (L.) Nábělek	Per	Ch	*+*	*+*	*-*	*-*	*+*	*-*	*-*	*-*	*-*	*-*	-
	*Scrophularia deserti* Delile	Ann	Th	*+*	-	-	-	-	-	-	-	-	-	-
Solanaceae	*Lycium shawii* Roem. & Schult.	Per	Ph	*+*	*+*	*-*	*-*	*+*	*-*	*+*	-	-	-	-
	*Hyoscyamus boveanus* (Dunal.) Asch. & Schweinf.	Per	Ch	*+*	*+*	*-*	*-*	*-*	*-*	*-*	*-*	*-*	*-*	-
	*H. desertorum* (Asch. ex Boiss.) Täckh.	Ann	Th	*+*	*-*	*-*	*-*	*+*	*-*	*-*	*-*	*-*	*-*	-
	*H. muticus* L.	Per	Ch	*+*	*-*	*-*	*-*	*-*	*-*	*-*	*-*	*-*	*-*	-
	**Solanum lycopersicum L.	Ann	Th	*-*	*-*	*-*	*-*	*-*	*-*	*-*	*-*	*+*	*-*	-
	*S. nigrum L.*	Ann	Th	*-*	*-*	*-*	*-*	*-*	*-*	*-*	*-*	*+*	*-*	-
Tamaricaceae	*Tamarix aphylla* (L.) H. Karst.	Per	Ph	*-*	*-*	*-*	*+*	*-*	*-*	*-*	*+*	*+*	*-*	
	*T. nilotica* (Ehrenb.) Bunge.	Per	Ph	*+*	*+*	*+*	*+*	*+*	*+*	*+*	*+*	*+*	*+*	
	*T. tetragyna* Ehrenb.	Per	Ph	*+*	*-*	*+*	*-*	*-*	*-*	*-*	*+*	*-*	*+*	
Urticaceae	*Forsskaolea tenacissima* L.	Per	Ch	*+*	-	-	-	*+*	*+*	*-*	*+*	*-*	*-*	*-*
Zygophyllaceae	*Tribulus mollis Ehrenb. ex Schweinf.	Ann	Th	*+*	-	-	-	-	-	-	-	-	-	-
	*T. terrestris* L.	Ann	Th	-	-	-	-	-	-	-	-	-	-	-
	*Zygophyllum album* L.	Per	Ch	*+*	-	-	-	-	-	-	-	*+*	-	-
	*Z. arabicum* (L.) Christenh. & Byng	Per	Ch	*+*	*+*	*-*	*+*	*+*	*-*	*-*	*-*	*-*	*-*	*-*
	*Z. bruguieri* (DC.) Christenh. & Byng	Per	Ch	*-*	*+*	-	-	-	-	-	-	-	-	-
	*Z. coccineum* L.	Per	Ch	*+*	*+*	*+*	*+*	*+*	*+*	*+*	*+*	*+*	*+*	*+*
	*Z. decumbens* Delile	Per	Ch	*+*	*+*	*+*								
	*Z. molle* (Delile) Christenh. & Byng	Per	Ch	*+*	*+*	-	-	*+*	-	-	-	-	-	-
	*Z. Simplex* L.	Ann	Th	*+*	*+*	*-*	*+*	*-*	*+*	*-*	*-*	*-*	*-*	*-*

*Note*: Duration: Ann = annual, Bi = biennial, Per = perennial. Life form: Th = Therophytes, H = Hemicryptophytes, Ph = phanerophytes, Ch = chamaephytes, C = Cryptophytes, P = Parasites, Asterisk (*) refers to new recorded species to the study regions, (**) refers to the species that was not mentioned in the checklist of Boulos (2009)

( + = recorded, - = not recorded)

TWINSPAN has categorized the collection sites in the current study into five main assemblages: *Zygophyllum coccineum – Halophyllum salicornicum assemblage*,* Zilla spinosa – Zygophyllum coccineum assemblage*,* Zygophyllum coccineum – Tamarix nilotica assemblage*,* Tamarix nilotica – Phargmites australis assemblage* and *Tamarix nilotica – Chenopodium murale* assemblage. The presence percentages of species composition for these assemblages are given (Table 2). It is clear that assemblage I has no species with 100% presence, but the highest ones are 72.8% (*Zygophyllum coccineum* and *Haloxylon salicornicum)*, and average abundance represents 3.5 and 2.88. So, they are the dominant species in this assemblage, followed by two codominant species, *Ochradenus baccatus* and *Farsetia aegyptia*, whose presence and average abundance represent 63.7%, 3.00 and 2.14. While *Anabasis setifera* and *Zilla spinosa* represent the associated species whose presence and average abundance represent 54.6%, 3.00, and 2.5. In the second assemblage, the dominant species with the highest presence 83.5%, 80.16% are *Zilla spinosa* and *Zygophyllum coccineum* and average abundance represent 3.16, 3.24. The codominant species is *Ochradenus baccatus*, whose presence and average abundance are 73.48% and 1.87. Moreover, *Launaea spinosa*,* Iphiona mucronata*, and *Echinops spinosus* represent the associated species, with 43.42%, 53.44%, and 40.8%, respectively, with an average abundance of 2.84, 2.62, and 2.5. In the third assemblage, the species with the highest presence are 74.2% *Zygophyllum coccineum* and *Tamarix nilotica*, and their average abundances represent 3.00 and 2.55. Followed by two codominant species, which are *Zilla spinosa* and *Ochradenus baccatus*, with a presence 59.36%, 51.94%, and an average abundance of 2.56, 3.00. While the associated species is represented by *Haloxylon salicornicum* whose presence and average abundance represent 37.1%, 2.2. The fourth assemblage has two species with 88% presence *Tamarix nilotica* and *Phargmites australis*, with average abundance 3.75, 3.12. Moreover, *Zygophyllum coccineum* represents the codominant species which represent 55.6%, 3.4 and the associated species is *Nitraria retusa* with a presence 33.36% and an average abundance 2.00. While the last assemblage includes six species with 100% presence e.g., *Bassia indica*, *Tamarix nilotica*, *Tamarix tetragyna*,* Chenopodium murale*,* Portulaca oleracea*, and *Sonchus oleraceus* with average abundance 3.00, 4.00, 1.00, 2.00, 2.00 respectively.

**Table 2:** Phytosociological table showing presence percentage and average abundance for main assemblages resulted from TWINSPAN classification. Avg = average abundance, P = Presence

Group	I		II		III		IV		V		Abbreviations
Species /No. of sites	11		30		27		9		2		
	*P*	Avg	*P*	Avg	*P*	Avg	*P*	Avg	*P*	Avg	
*Achillea fragrantissima*			16.7	1.6							*Achi fra*
*Aerva javanica*							11.12	2.00			*Aerv jav*
*Aizoon canariense*			3.34	1.00							*Aizo can*
*Alhagi graecorum*					3.71	2.00	22.24	3.5			*Alha gra*
*Amaranthus viridis*							11.12	2.00	50	2.00	*Amar vir*
*Anabasis articulata*	9.1	2.00									*Anab art*
*Anabasis setifera*	54.6	3.00	6.68	1.5	3.71	2.00					*Anab seti*
*Anastatica hierochuntica*	18.4	1.5	13.36	1.00	3.71	1.00					*Anas hie*
*Artemisia judaica*			3.34	1.00							*Arte jud*
*Artemisia monosperma*					3.71	2.00					*Arte mon*
*Arthrocaulon macrostachyum*					11.13	2.00					*Arth mac*
*Asphodelus tenuifolius*			3.34	3.00							*Asph ten*
*Astragalus spinosus*						11.12	1.00				*Astr spi*
*Astragalus sieberi*			3.34	2.00							*Astr sie*
*Atriplex humilis*			3.34	1.00	3.71	1.00					*Atri hum*
*Bassia eriophora*			3.34	2.00			11.12	2.00			*Bass eri*
*Bassia indica*	9.1	2			14.84	3.00			100	3.00	*Bass ind*
*Bassia muricata*					3.71	2.00	11.12	2.00			*Bass mur*
*Blepharis edulis*	9.1	1.00									*Blep edu*
*Brocchia cinerea*			3.34	2.00							*Broc cin*
*Calligonum comosum*			10.02	3.00							*Call com*
*Calotropis procera*			10.02	2.66	22.26	2.33					*Calo pro*
*Caroxylon imbricatum*	9.1	1.00	3.34	1.00							*Caro imb*
*Caroxylon inerme*					3.71	3.00					*Caro ine*
*Caylusea hexagyna*	9.1	3.00									*Cayl hex*
*Cenchrus biflorus*									50	2.00	*Cenc bif*
*Cenchrus divisus*					3.71	2.00					*Cenc div*
*Centaurea aegyptiaca*			6.68	1.5	7.42	2.00					*Cent aeg*
*Centaurea pallescens*			16.7	2.00							*Cent pal*
*Chenopodium album*									50	2.00	*Chen alb*
*Chenopodium ficifolium*									50	2.00	*Chen fic*
*Chenopodium murale*									100	4.00	*Chen mur*
*Cistanche tubulosa*	9.1	2.00									*Cist tub*
*Citrullus colocynthis*	9.1	2.00	16.7	1.6	3.71	2.00					*Citr col*
*Cleome amblyocarpa*					11.13	2.66					*Cleo amb*
*Cleome droserifolia*			3.34	2.00	3.71	1.00	11.12	2.00			*Cleo dro*
*Coincya tournefortii*					3.71	2.00					*Coin tou*
*Convolvulus hystrix*			13.36	2.5	3.71	1.00					*Conv hys*
*Convolvulus lanatus*			6.68	1.00	16.85	2.00					*Conv lan*
*Crotalaria aegyptiaca*			40.08	2.41							*Crot aeg*
*Cucumis melo*					3.71	1.00					*Cucu mel*
*Cynanchum acutum*			3.34	2.00	7.42	0.14			50	2.00	*Cyna acu*
*Cynodon dactylon*					3.71	3.00			50	2.00	*Cyno dac*
*Deverra tortuosa*	9.10	2.00	10.2	2.6			11.12	1.00			*Deve tor*
*Diplachne fusca.*					3.71	1.00					*Dipl acr*
*Diplotaxis acris*					3.71	3.00					*Dipl fus*
*Diplotaxis harra*					7.42	2.00					*Dipl har*
*Echinops spinosus*	9.1	3.00	40.08	2.5							*Echi spi*
*Ephedra alata*			3.34	1.00							*Ephe ala*
*Eremobium aegyptiacum.*	18.2	2.00									*Erem aeg*
*Erigeron bonariensis*			3.34	3.00					50	3.00	*Erig bon*
*Euphorbia peplus*									50	2.00	*Euph pep*
*Euphorbia retusa*			3.34	2.00	3.71	2.00					*Euph ret*
*Farsetia aegyptia*	63.7	3.00	6.68	1.5	3.71	2.00					*Fars aeg*
*Forsskaolea tenacissima*			6.68	2.00	11.13	3.66					*Fors ten*
*Gymnocarpos decander*	18.2	2.00									*Gymn dec*
*Gypsophila capillaris*			6.68	3.00							*Gyps cap*
*Halopeplis perfoliata*					3.71	1.00	11.12	2.00			*Halo per*
*Haloxylon persicum*							11.12	2.00			*Halo sal*
*Haloxylon salicornicum*	72.8	2.88	40.08	2.25	37.1	2.2	11.12	1.00			*Halo sco*
*Haloxylon scoparium*	18.2	2.00									*Halop per*
*Haplophyllum tuberculatum*			6.68	1.5							*Hapl tub*
*Heliotropium arbainense*			23.38	1.57							*Heli arb*
*Heliotropium bacciferum*					7.42	2.00					*Heli bac*
*Heliotropium digynum*			3.34	2.00							*Heli dig*
*Hyoscyamus boveanus*			10.02	3.00	3.71	2.00					*Hyos bov*
*Hyoscyamus desertorum*			6.68	3.00							*Hyos des*
*Hyoscyamus muticus*	9.1	1.00	3.34	4.00	7.42	2.00					*Hyos mut*
*Ifloga spicata*			6.68	2.00							*Iflo spi*
*Imperata cylindrica*					3.71	2.00	11.12	4.00			*Impe cyl*
*Iphiona mucronata*			53.44	2.62	3.71	1.00					*Iphi muc*
*Ipomoea pes-caprae*					3.71	1.00					*Ipom pes*
*Juncus rigidus*							11.12	4.00			*Junu rig*
*Kickxia aegyptiaca*	9.1	2.00	10.02	2.00							*Kick aeg*
*Lasiurus scindicus*	9.1	2.00	13.36	1.4	27.42	1.5					*Lasi sci*
*Launaea nudicaulis*				10.02	1.66						*Laun muc*
*Launaea procumbens*					3.71	2.00					*Laun nud*
*Launaea spinosa*			43.42	2.84	3.71	1.00					*Laun pro*
*Launaea mucronata*			10.02	2.00	22.26	1.57					*Laun spi*
*Lavandula coronopifolia.*			16.70	1.6							*Lava cor*
*Lepidium didymum*									50	2.00	*Lepi did*
*Leptadenia pyrotechnica*	18.2	3.00	0.43	2.14	14.84	1.00					*Lept pyr*
*Limonium pruinosum*					3.71	1.00					*Limo pru*
*Lycium shawii*	45.5	2.4	26.72	2.00	3.71	1.00					*Lyci sha*
*Lysimachia arvensis*									50	2.00	*Lysi arv*
*Malva parviflora*									50	3.00	*Malv par*
*Matthiola longipetala*	9.1	2.00									*Matt l bi*
*Matthiola longipetala*	9.1	2.00									*Matt l liv*
*Melilotus indicus*							11.12	4.00			*Meli ind*
*Monsonia nivea*					3.71	1.00					*Mon niv*
*Neurada procumbens*			3.34	1.00							*Neur pro*
*Nidorella aegyptiaca*			3.34	2.00	3.71	1.00					*Nido aeg*
*Nitraria retusa*			3.34	2.00	7.42	2.5	33.36	2.00			*Nitr ret*
*Ochradenus baccatus*	63.7	2.14	73.48	1.87	51.94	3.00	11.12	3.00			*Ochr bac*
*Panicum turgidum*	9.1	2.00	30.06	2.55	7.42	1.5					*Pani tur*
*Paronychia sinaica*			3.34	1.00							*Paro sin*
*Pergularia tomentosa*			16.7	2.00							*Perg tom*
*Phoenix dactylifera*							22.24	1.5			*Phoe dac*
*Phragmites australis*			6.68	2.00	7.42	3.00	88.96	3.12			*Phra aus*
*Plantago ovata*			3.34	1.00							*Plat ova*
*Pluchea dioscoridis*			6.68	2.00	7.42	2.00					*Pluc dio*
*Portulaca oleracea*									100	2.00	*Port ole*
*Pulicaria incisa*			3.34	2.00	22.26	1.66					*Puli inc*
*Pulicaria undulata*	6.68	2.5	10.02	2.33	11.13	2.33					*Puli und*
*Reichardia tingitana*					3.71	2.00					*Reic tin*
*Reseda pruinosa*					3.71	3.00					*Rese pru*
*Retama raetam*	9.1	2.00	6.68	2.00							*Reta rae*
*Rumex cyprius*					3.71	1.00					*Rume cyp*
*Rumex spinosus*					3.71	1.00					*Rume spi*
*Rumex vesicarius*	9.1	1.00			14.84	1.75					*Rume ves*
*Salvia aegyptiaca*			3.34	1.00							*Salv aeg*
*Scrophularia deserti*			3.34	2.00							*Scro des*
*Senecio glaucus*					7.42	1.5					*Sene gla*
*Solanum lycopersicum*									50	3.00	*Sola lyc*
*Solanum nigrum*									50	2.00	*Sola nig*
*Sonchus oleraceus*			3.34	1.00	3.71	2.00			100	2.00	*Sonc ole*
*Tamarix aphylla*			3.34	4.00	3.71	1	11.12	2.00			*Tam aph*
*Tamarix nilotica*			26.72	2.5	74.2	2.55	88.92	3.75	100	4.00	*Tam nil*
*Tamarix tetragyna*			3.34	4.00	74.2	2.00	11.12	2.00	100	1.00	*Tam tet*
*Taverniera aegyptiaca*					7.42	1.5	11.12	2.00			*Tave aeg*
*Traganum nudatum*			3.34	2.00	18.55	2.00					*Trag nud*
*Tribulus mollis*			6.68	1.5							*Trib mol*
*Tribulus terrestris*					3.71	2.00					*Trib ter*
*Trichodesma africanum*			13.36	2.00	18.55	2.8	11.12	1.00			*Tric afr*
*Tricholaena teneriffae*	9.1	2.00					11.12	2.00			*Tric ten*
*Urospermum picroides*									50	1.00	*Uros pic*
*Vachellia tortilis subsp. raddiana*	27.3	1.66	20.04	0.3	3.71	1.00					*Vac t rad*
*Vachellia tortilis subsp. tortilis*	9.1	1.00	6.68	1.5	3.71	1.00					*Vac t tor*
*Zilla spinosa*	54.6	2.5	83.5	3.16	59.36	2.56					*Zill spi*
*Zygophyllum coccineum*	72.8	3.5	80.16	3.24	74.2	3.00	55.6	3.4	50	4.00	*Zyg ara*
*Zygophyllum arabicum*	18.2	1.5	3.34	2.00	25.97	1.42					*Zyg coc*
*Zygophyllum bruguieri*	9.1	1.00			3.71	2.00					*Zyg bru*
*Zygophyllum album*			3.34	2.00			11.12	2.00			*Zyg alb*
*Zygophyllum decumbens*			30.06	1.77	7.42	2.00					*Zyg dec*
*Zygophyllum molle*	27.3	2.33	13.36	1.75	7.42	2.00					*Zyg mol*
*Zygophyllum Simplex*	18.2	2.00	3.34	1.00	14.84	2.25	11.12	2.00			*Zyg Sim*

This study included sixteen physical and chemical environmental factors. These factors can be classified into two main groups. The first group is a physical characteristic of the soil and soil texture by pipette method, soil moisture content, organic carbon, and organic matter content. The second group, which is the chemical characteristics of soil, includes acidity (pH), electric conductivity (EC), cations, anions, and total carbonate, which is given as a percentage of weight. The ranges and means of the environmental variables of sites supporting each assemblage reveal a general idea about the magnitude of variation in the environmental factors in the study area. There are some differences between the five assemblages’ physical attributes. The largest percentages of clay (28.2%), organic matter (1.18%), organic carbon (0.68%), and soil moisture content (12.1%) are found in the fourth assemblage. Silt percentages are highest in assemblage one at 60.5%, whereas the largest values of sand are found in the second assemblage, which shows 92.5% of the total. (Table 3)

**Table 3:** Minimum, maximum, mean, and standard deviation of environmental variables for the main assemblages resulted from TWINSPAN

Assemblage	I				II				III				IV				V			
No. of sites	11				30				27				9				2			
	Min	Max	Mean	Std Dev	Min	Max	Mean	Std Dev	Min	Max	Mean	Std Dev	Min	Max	Mean	Std Dev	Min	Max	Mean	Std Dev
Physical properties																				
Soil texture (pipette)																				
Sand%	29	90.5	62.6	17.54	34.1	92.5	72.19	12.09	37.5	85.8	65.46	11.32	5.22	56.3	80.5	69.78	74.4	82.9	78.65	6.01
Silt%	3.7	60.5	17	16.63	2.2	27.3	9.48	6.37	4.8	37.5	14.72	7.47	7.3	21.6	14.8	5.10	9.3	9.8	9.55	0.35
Caly%	5.8	23.8	12.94	5.55	1	30	9.36	5.70	3	22.7	12.53	5.22	3.05	28.2	12.99	8.46	7.3	16.3	11.8	6.36
Organic matter%	0.05	0.61	0.23	0.18	0.054	0.74	0.32	0.18	0.05	1.11	0.35	0.32	0.08	1.18	0.37	0.33	0.10	0.38	0.24	0.19
Organic carbon %	0.31	0.35	0.138	0.107	0.031	0.43	0.031	0.43	0.03	0.64	0.20	0.188	0.04	0.68	0.19	0.21	0.06	0.22	0.14	0.11
Moisture content %	0.26	3.9	1.27	0.98	0.4	11.5	2.15	2.22	0.2	0.2	1.76	1.30	0.4	12.1	4.41	3.67	2.5	11.9	7.2	6.64
Chemical properties																				
pH	7.77	9.24	8.63	0.48	7.61	7.61	7.61	5.42	7.13	8.55	7.94	0.37	6.87	8.24	7.68	0.44	0.06	8.47	8.26	0.28
EC (dsm^− 1^)	1.3	10.8	5.89	3.03	1.06	62.6	1.06	62.6	1.4	76.2	15.56	19.97	4.83	181	74.59	64.73	8.14	27.8	17.97	13.90
Cations ( mEq/L )																				
Ca^2+^	7	33	21.97	8.35	2.8	84	2.8	84	7	240	55.10	63.03	33	470	140.28	138.31	15.3	59.7	37.5	31.39
Mg^2+^	5.6	22	14.53	5.28	2.1	44	13.56	89.11	5	150	32.15	38.96	10.4	180	77.91	57.97	14.5	47	30.75	22.98
Na^+^	3.2	56.3	23.87	20.77	3.1	497	13.41	7.53	4.4	378	68.36	99.06	4.6	1496	526.28	497.33	51.3	171	115.11	84.64
K^+^	0.1	0.5	0.29	0.14	0.2	1	0.30	0.16	0.2	2	0.46	0.53	0.3	3	1.42	0.97	0.3	0.5	0.4	0.14
Anions ( mEq/L )																				
HCO3^−^	1.8	12.2	6.67	3.55	1.4	25	6.98	5.27	1.6	71	12.18	17.02	9	81	34.4	26.90	11.2	19.7	15.45	6.01
Cl^−^	6.2	69	31.19	18.11	5.1	398	39.09	69.21	5.5	496	94.98	127.18	28	1410	529.11	508.93	34.9	160	97.45	88.45
SO4^2−^	4.6	39.7	11.57	21.03	3.3	203	23.51	43.91	4.7	217	48.48	57.69	11.3	335	182.43	130.90	35.3	98.2	66.75	44.47
CaCO3%	7.5	32.3	20.86	8.86	7.6	34.3	19.86	7.22	6.5	33.2	14.68	6.90	8	24.9	18.02	6.02	22.5	23.3	22.9	0.56

While in the case of the chemical properties, it’s obvious that the maximum values of EC, Na^+^, Mg^2+^, K^+,^ Ca^2+^, Cl^−,^ HCO_3_^−^ and SO_4_^2−^ contributed to the fourth assemblage representing 181 dsm^− 1^., 1496 mEq/L, 180 mEq/L, 470 mEq/L, 3 mEq/L, 1410 mEq/L, 81 mEq/L and 182.43 mEq/L. On the other hand, the maximum value of pH and CaCO_3_ are represented by 9.24, 32.3% in the second assemblage.

**Fig. 6:**
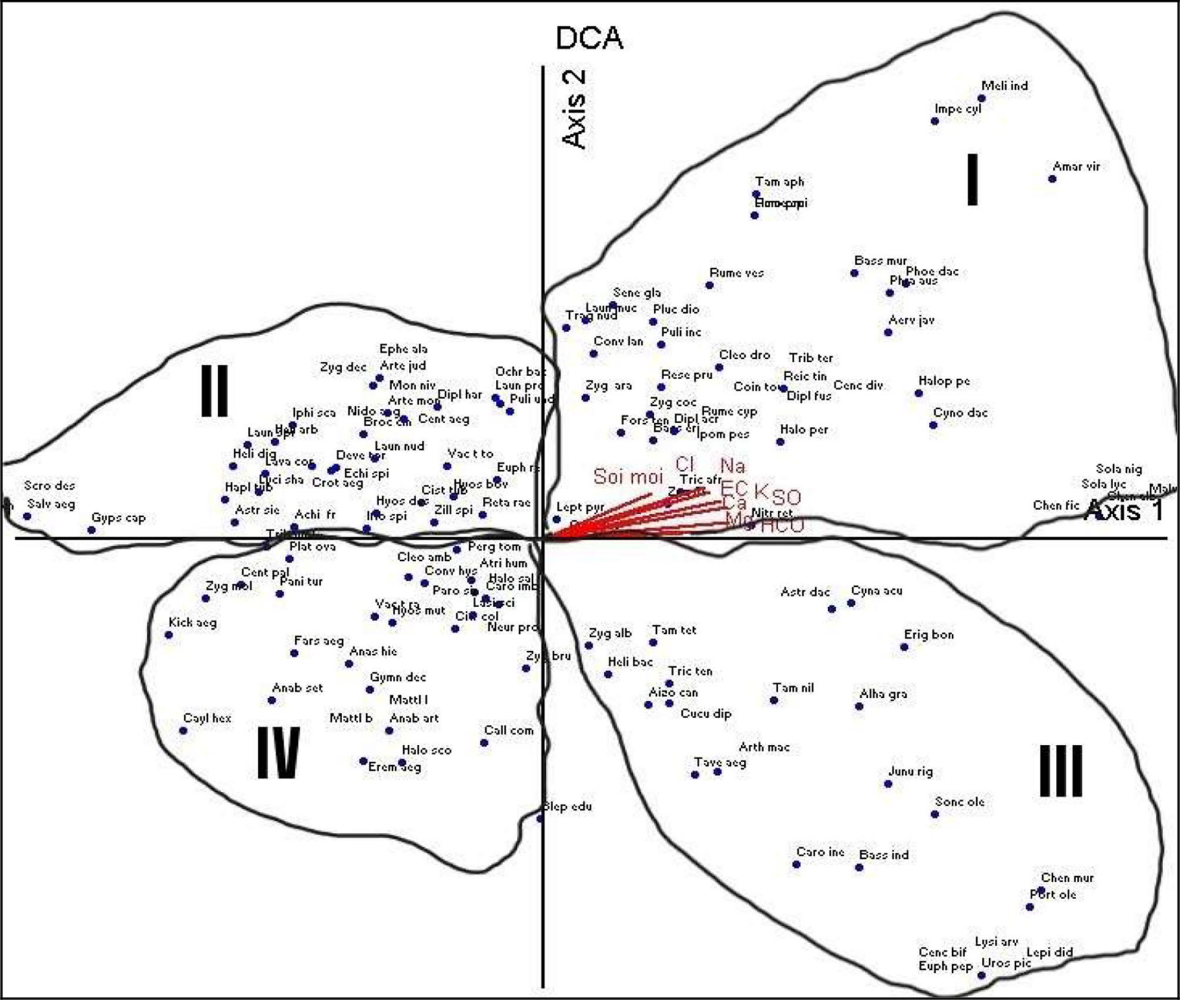
Ordination diagram (DCA) with plant species as points and selected environmental variables (physical and chemical)

The DCA ordination showed that the 136 plants could be classified into four sections (Fig. 6). The first section (I) of plants was positively affected by the performed edaphic factors, including the following plants: *Nitraria retusa*,* Trichodesma africanum*,* Zygophllum simplex*,* Zygophyllum coccineum*,* cleome droserifolia* and *melilotus indicus. Pergularia tomentosa*,* Cleome amplycarpa*,* Panicum turgidum*,* Zygophyllum molle* and *Caylusea hexagyna* represented the fourth section (IV) negatively affected by edaphic factors. Then, the second section (II) included *Zilla spinosa*,* Launaea nudicaulis and Iphiona mucronata* affected by the physical and chemical factors but less than the first and the fourth section. Moreover, the third section (III) represented by *Zygophyllum album*,* Aizoon canariense*,* Bassia indica* and *Portulaca oleracea* was the same as the second one in its relation with edaphic factors. Nevertheless, because *Blepharis edulis* lied on the axis, the applied edaphic factors had no influence.

## Discussion

Although, 136 species were found in the current investigation. The species number in the study could be more than the given number. However, extremely dry weather has a diverse impact on the development and growth of several species. In addition, the quarries, cement industries, and summer resorts devastate the natural habitats of the wild plants.

By comparing the most abundant plants of this study with those of Danin [5], Mashaly [6], and Zahran and Willis [7], it becomes clear that several species have undergone significant changes in their presence. *Ephedra alata*,* Blepharis edulis*,* Hyparrhenia hirta*,* Phagnalon barbeyanum*,* Reaumuria hirtella*,* Anastatica hierochuntica*,* Verbena officinalis*, and *Achillea santolina*, which were dominant plants in the study area, have experienced a dramatic decline in population. These species are now facing local extinction or severe scarcity which may due to the combined effects of human activities and natural climatic change.

Furthermore, because of extensive overgrazing, overcollection, overcutting, and uprooting by locals and herbalists for research, fuel, medicinal uses, and local trade, other plants like *Artemisia Judaica*,* Artemisia monosperma*,* Cleome droserifolia*,* Haplophyllum tuberculatum*,* and Cotula cinerea* become extremely rare [7].

**Fig. 7:**
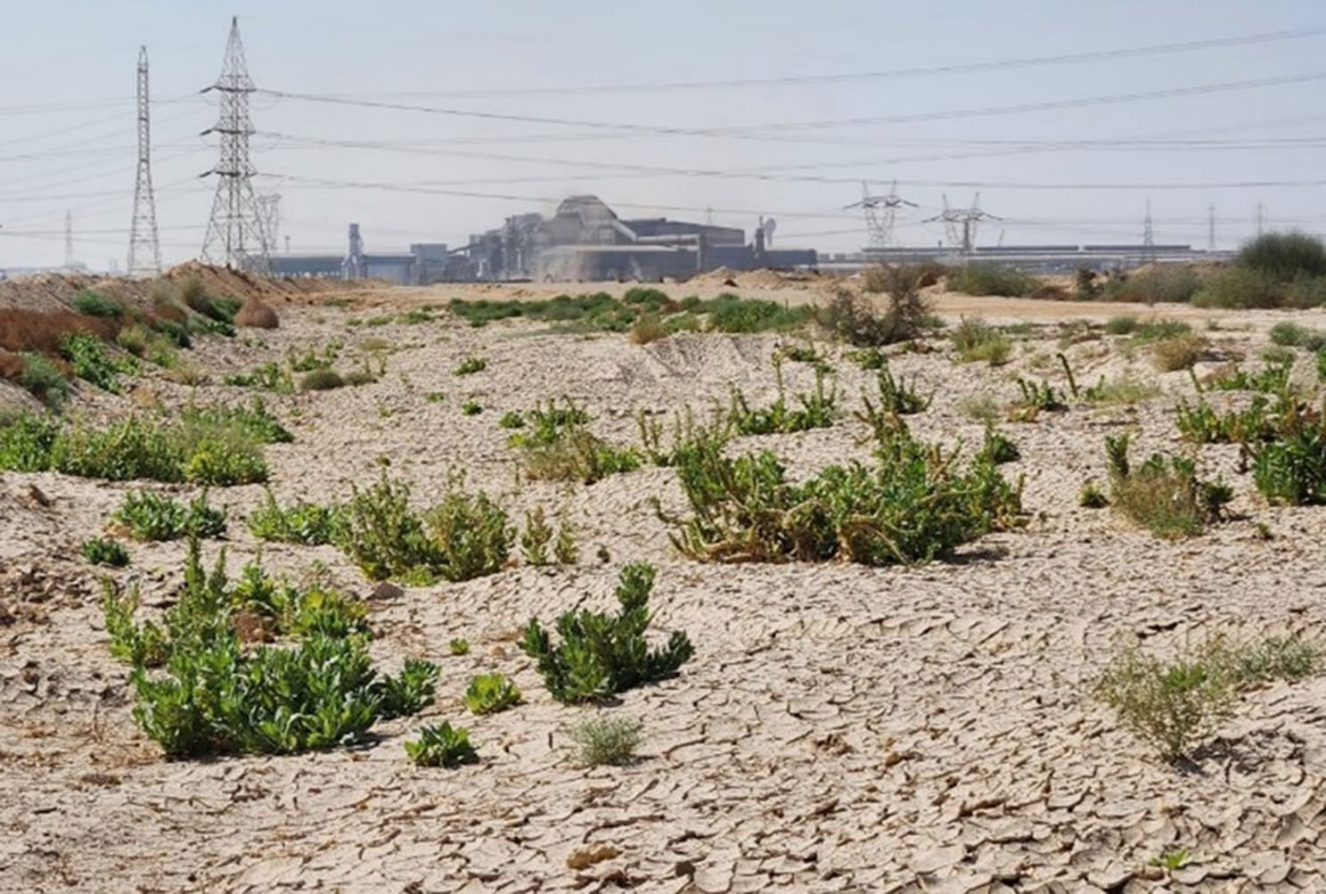
Showing the high cover of *Hyoscyamus muticus* in some sites of Wadi Hagul

Abdelaal [10] and Bedair et al. [13] stated that *Hyoscyamus muticus* is a threatened and rare plant in Wadi Hagul. However, in this study, it wasn’t rare, and its cover could reach 70% and 50% in some sites of the Wadi (Fig. 7).

No study has comprehensively covered the entire region, but several studies have focused on limited sites within the study area, such as Wadi Hagul. This study revealed that Wadi Hagul included 88 species, which was higher than that given by Bedair et al. [13] (80 species), Khdery et al. [11] (27 species), Mashaly et al. [31] (57 species), and Mohamed [8] (82 species). Bedair et al. [13] shared with this study 75 species, while many others shared 27 species, 53 species, and 61 species. In contrast to Abdelaal [10] recorded 98 species sharing 73 species out of 88 species.

In the study region, the three families with the highest number of species were Asteraceae (16.1%), Amaranthaceae (13.9%), and Brassicaceae (7.3%). The order of these families does not match any of the previously mentioned Wadi Hagul studies. However, all of them confirmed that Asteraceae is the largest family in species numbers given in this study.

Because of the high levels of aridity and salinity in this area, the main families are Asteraceae and Amaranthaceae, which are remarkable for having xerophytic species and for their high ability of salt tolerance [32]. Moreover, Judd and Ferguson [33] confirmed that Amaranthaceae predilated semi-arid climates and saline habitats.

The recorded species in the study area were represented by 61.7% perennials and 37.76% annuals. This dominance of perennials was confirmed by Bedair et al. [13], Khdery et al. [11], Mashaly et al. [31] and Mohamed [8]. The possible explanation for the predominance of perennials is the little rainfall there.

The life forms spectrum of Gebel Ataqa region is dominated by therophytes and chamaephytes (38.2%) followed by phanerophytes (11%) hemicryptophytes (8.8%), cryptophytes (2.9%), the latter agreed with Khdery et al. [11].

Therophytes and chamaephytes dominance is a sign of human influence, hot, dry climate with little rainfall, and the lack of readily available microhabitats in the area that may support a large percentage of perennials [34]. While the highest percentages of chamaephytes and hemicryptophytes seem to be a tool of adaptation against drought, salinity, sand accumulation, and grazing [35].

It is evident by comparing the flora of the investigated wadis that Wadi Hagul had the largest number of species. Wadi Hagul is considered to be an attractive environment for various plants, including a number of endangered and threatened ones, more than any other wadis in the study area.

In response to physiographic features, harsh climatic conditions, and human activities, specific plant communities, species composition, variety, and cover are bioindicators for the consistency and conservation status assessment of desert ecosystems [10].In the current study, the collection sites are classified by TWINSPAN to five main assemblages namely; *Zygophyllum coccineum –Haloxylon salicornicum* assemblage, *Zilla spinosa - Zygophyllum coccineum* assemblage, *Zygophyllum coccineum-Tamarix nilotica* assemblage, *Tamarix nilotica – Phargmites australis* assemblage and *Tamarix nilotica – Chenopodium murale* assemblage. These assemblages agreed somehow. These assemblages agreed somehow with Abdelaal [10] and Mashaly [6] as they showed the dominance of *Zygophllum coccineum* and *Zilla spinosa* in their clusters.

The plants assemblages of the current study varied from those of the others because they targeted Wadi Hagul only, representing 41% of this study area of this study.

The DCA ordination demonstrated that the distribution of plants was impacted in both positive and negative ways by physical and chemical analysis, and this agreed Mashaly [6].

*Zygophyllum coccineum* dominated on three assemblages due to its amazing ability to inhabit a wide range of soil types and occupy various habitats. In the plains and limestone wadies of the Eastern desert, the plant is widely distributed and tolerant of saline soils [36]. Moreover, *Zilla spinosa* is tolerant of drought, even during extremely hot months. Because it tends to raise its soluble sugar content in response to heat or water stress, producing a significant amount of osmotic potential [37]. So, their high tolerance agrees to the high silt, sand, and CaCO3% level that represented the first and second assemblage**s.**

**Fig. 8:**
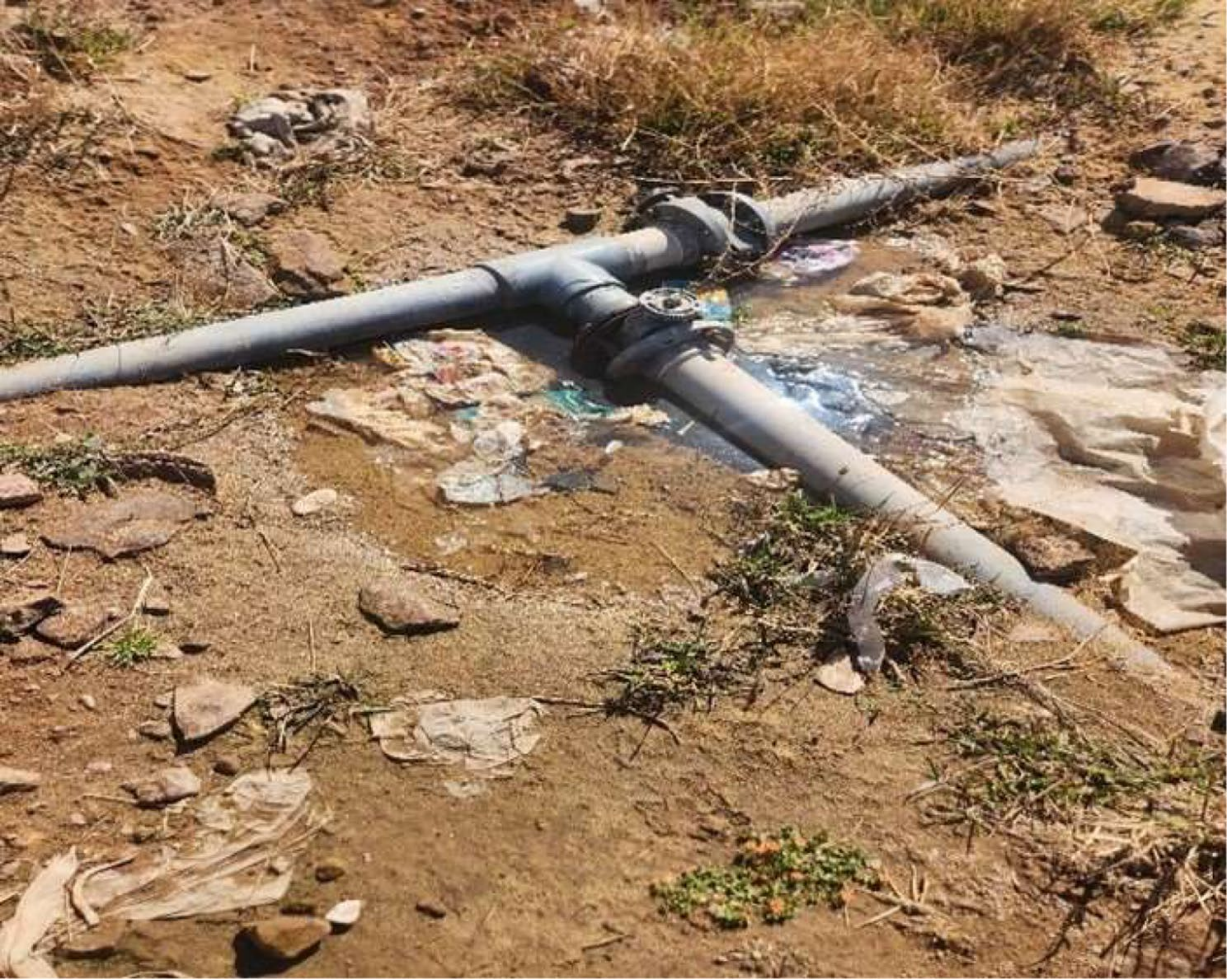
Showing the source of water that led to the presence of introduced species to wadi

*Tamarix nilotica* dominated two assemblages and *Phargmites australis* dominated only one assemblage due to their salt and drought tolerance [9]. Thus, their high resistance to drought and salinity corresponds to the highest values of EC, which represents the fourth assemblage. On the other hand, the study area displayed the recruitment of some introduced species in very limited presence, such as *Chenopodium murale*, *Chenopodium album*,* Chenopodium ficifolium*,* Cenchrus biflorus*, *Malva parviflora*, *Portulaca oleracea*, *Melilotus indicus* and *Lysimachia arvensis*. The reason for this is the presence of a water source very near them (Fig. 8). Moreover, their presence is dangerous for biodiversity and natural resources, especially in dry environments. Effective invaders frequently show adaptation, flourishing in a variety of habitats. Features of the plant shoot and root system are considered morphological requirements for the invasion to be successful in different environments. Moreover, allelopathic chemicals are found in many invasive species, which allow them to dominate plant populations [13].

It must be noted that these introduced species are absent in Wadi Hagul and neighboring wadis as the climate of these wadis is hyper-arid, and rainfall was very rare during the years of plant collection excursions which reflect the impact of climate change So, these results disagreed with those of Bedair et al. [13], Abdelaal [10] and agreed with Khdery et al. [11].

*Cucumis melo* and *Solanum lycopersicum* are invasive species to the Egyptian flora that has escaped from its native habitat and are growing naturally in the local flora. It has been unintentionally transferred from one area to another by humans [38], reflecting the bad impact of human interference on the native flora.

In this study, *Tribulus mollis* is added for the first time to the flora of Eastern desert flora, so its geographical distribution disagreed Boulos [24] who mentioned that it is located only in Sinai.

## Conclusion

This study acts as evidence of the destructive impact of anthropogenic hazards (quarries, construction of roads, cement factories, summer resorts, over-collection) and a hyper-arid climate on the flora of unprotected mountainous areas facing the north-east section of the Gulf of Suez, causing it to change or disappear. These threats are also causing an increase in introduced and invasive species, which negatively impacts the density of native populations. With the help of the information obtained from this study and others about the current floristic composition losses, threats to the ecosystem, and the spatial distribution of plant communities, an effective conservation strategy and plan can be developed to try to recover and restore the endangered desert ecosystem.

## Electronic supplementary material

Below is the link to the electronic supplementary material.

Supplementary Material 1

## Data Availability

The datasets of this study are available from the corresponding author upon request.
