# Supplementary material for: Floral diversity and conservation status of vascular plants in arid mountainous areas
Source: BMC Ecol Evol. 2025 Jan 7;25:3. doi: 10.1186/s12862-024-02342-y (PMC11705987; doi:10.1186/s12862-024-02342-y)
Supplement: Supplementary file 1 — Supplementary Material 1 [file 12862_2024_2342_MOESM1_ESM.docx]

**Table (1): Information about the collected samples from the study area**.

| **No. of sites** | **Site** | **GPS**  **data** |
| --- | --- | --- |
| **1** | Wadi Hemra | 30° 04' 08"N  32°22' 36"E  E 112m |
| **2** | Wadi Hemra | 30° 04' 08"N  32°22' 32"E  E 113m |
| **3** | Wadi Hemra | 30° 05' 47"N  32°18' 14"E  E 171m |
| **4** | Wadi Hemra | 30° 05' 47"N  32°17' 54"E  E 185m |
| **5** | Cairo-Suez Road | 30° 04' 08"N  32°06' 17"E  E 278m |
| **6** | Cairo-Suez Road | 30° 04' 09"N  32°06' 18"E  E 279m |
| **7** | Wadi Hagul | 30° 04' 24"N  32°05' 52"E  E 280m |
| **8** | Wadi Hagul | 29° 56' 22"N  32°10' 59"E  E 330m |
| **9** | Wadi Hagul | 29° 55' 26"N  32°12' 14"E  E 654m |
| **10** | Wadi Hagul | 29° 56' 22"N  32°10' 59"E  E 338m |
| **11** | Wadi Hagul | 29° 53' 1"N  32°15' 33"E  E 550m |
| **12** | Wadi Hagul | 29°51'7"N  32°15'16"E  622 |
| **13** | Wadi Hagul | 29° 49' 29"N  32°16' 11"E  E 654m |
| **14** | Wadi El-Bada | 29° 44' 46"N  32°14' 11"E  E 85m |
| **15** | Wadi El-Bada | 29° 44' 46"N  32°14' 11"E  E 85m |
| **16** | Wadi El-Bada | 29° 45' 36"N  32°13' 28"E  E110m |
| **17** | Wadi El-Bada | 29° 46' 08"N  32°12' 01"E  E 114m |
| **18** | Between Wadi El-Bada and GebelUm Zeita | 29° 45' 51"N  32°10' 18"E  E 145m |
| **19** | GebelEl-Ramliya | 29° 46' 43"N  32°08 14"E  E 152m |
| **20** | Wadi El-Gamil | 29° 49' 25"N  32°28' 41"E  E 5m |
| **21** | Wadi El-Gamil | 29° 47' 23"N  32°26' 14"E  E 7m |
| **22** | Wadi El-Ramliya | 29° 47' 23"N  32°25' 54"E  E 10m |
| **23** | Wadi El-Ramliya | 29° 47' 17"N  32°25' 50"E  E 9m |
| **24** | Between Wadi Hagul and Wadi El-Ramliya | 29° 45' 13"N  32°23' 6"E  E 10m |
| **25** | Between Wadi Hagul and Wadi El-Ramliya | 29° 45' 09"N  32°23' 06"E  E 19m |
| **26** | Wadi Hagul | 29° 42' 30"N  32°20' 37"E  E 39m |
| **27** | Wadi Hagul | 29° 41' 25"N  32°20' 17"E  E 15m |
| **28** | Ain Shokna | 29° 35' 12"N  32°20' 34"E  E 19m |
| **29** | Wadi Amlog | 29° 32' 11"N  32°22' 12"E  E 9m |
| **30** | Wadi Amlog | 29° 31' 36"N  32°22' 52"E  E 12m |
| **31** | Wadi Hagul | 29° 58' 37"N  32°07' 29"E  E 312m |
| **32** | Wadi Hagul | 29° 56' 38"N  32°10' 46"E  E 312m |
| **33** | Wadi Hagul | 29° 54' 06"N  32°13' 7"E  E 208m |
| **34** | Wadi Hagul | 29° 53' 35"N  32°13' 51"E  E 92m |
| **35** | Wadi Hagul | 29° 53' 12"N  32°14' 20"E  E 29m |
| **36** | Wadi Hagul | 29° 48' 00"N  32°16' 43"E  E 102m |
| **37** | Wadi El-Bada | 29° 43' 20"N  32°16' 36"E  E 59m |
| **38** | Wadi El-Bada | 29° 42' 43"N  32°17' 8"E  E 37m |
| **39** | Wadi Hagul | 29° 47' 51"N  32°16' 35"E  E 115m |
| **40** | Wadi Hagul | 29° 47' 48"N  32°16' 37"E  E 102m |
| **41** | Infront of GebelMoghra Bahria | 29° 52' 16"N  32°28' 18"E  E 10m |
| **42** | Wadi El-Gamil | 29° 49' 25"N  32°28' 41"E  E 7m |
| **43** | Wadi El-Gamil | 29° 48' 07"N  32°27' 11"E  E 16m |
| **44** | Between Wadi Hagul and Wadi El-Ramliya | 29° 44' 15"N  32°22' 32"E  E 9m |
| **45** | Wadi Hagul | 29° 42' 30"N  32°20' 40"E  E 20m |
| **46** | Wadi Hagul | 29° 42' 32"N  32°20' 35"E  E 24m |
| **47** | Ain Shokna | 29° 35' 20"N  32°20' 12"E  E 53m |
| **48** | Infront of GebelUm Rosis | 29° 32' 22"N  32°22' 08"E  E 30m |
| **49** | Wadi Amlog | 29° 31' 56"N  32°22' 26"E  E 20m |
| **50** | Wadi Amlog | 29° 31' 58"N  32°22' 22"E  E 19m |
| **51** | Wadi Hagul | 29° 59' 50"N  32°05' 48"E  E 10m |
| **52** | Wadi Hagul | 30° 00' 01"N  32°05' 44"E  E 292m |
| **53** | Wadi Hagul | 29° 58' 08"N  32°08' 32"E  E 16m |
| **54** | Wadi Hagul | 29° 54' 21"N  32°12' 49"E  E 220m |
| **55** | Wadi Hagul | 29° 53' 52"N  32°13' 10"E  E 220m |
| **56** | Wadi Hagul | 29° 53' 52"N  32°13' 10"E  E 220m |
| **57** | Wadi El-Bada | 29° 46' 49"N  32°08' 22"E  E 148m |
| **58** | Infront of GebelUm Rosis | 29° 32' 22"N  32°22' 08"E  E 147m |
| **59** | Wadi El-Bada | 29° 46' 23"N  32°10' 10"E  E 125m |
| **60** | Between GebelEl-Akheider and  Wadi Ghweiba | 29° 42' 21"N  32°10' 14"E  E 124m |
| **61** | Wadi Hagul | 29° 42' 29"N  32°20' 39"E  E 10m |
| **62** | Wadi Hagul | 29° 42' 33"N  32°20' 36"E  E 9m |
| **63** | Wadi Abu Dahab | 29° 31' 27"N  32°23 55"E  E 9m |
| **64** | Infront of GebelMasama | 29° 30' 37"N  32°24' 01"E  E 7m |
| **65** | Near to Ras Abu Darag | 29° 21' 52"N  32°34' 12"E  E 35m |
| **66** | Near to Ras Abu Darag | 29° 21' 57"N  32°34' 14"E  E 26m |
| **67** | Ras Abu Darag | 29° 19' 34"N  32°35' 33"E  E 19m |
| **68** | Ras Abu Darag | 29° 19' 31"N  32°35' 31"E  E 36m |
| **69** | Wadi Malaha | 29° 15' 10"N  32°32' 08"E  E 12m |
| **70** | Wadi Khurri | 29° 14' 04"N  32°32' 19"E  E 12m |
| **71** | Wadi Hagul | 29° 58' 10"N  32°08' 23"E  E 338m |
| **72** | Wadi Hagul | 29° 53' 40"N  32°13' 23"E  E 215m |
| **73** | Wadi Hagul | 29° 49' 11"N  32°16 17"E  E 153m |
| **74** | Wadi Hagul | 29° 49' 12"N  32°16' 05"E  E 131m |
| **75** | Wadi Hagul | 29° 47' 49"N  32°16' 29"E  E 119m |
| **76** | Wadi Hagul | 29° 46' 15"N  32°12' 00"E  E 115m |
| **77** | Wadi El-Bada | 29° 46' 20"N  32°11' 15"E  E 125m |
| **78** | Wadi El-Bada | 29° 46' 49"N  32°07' 18"E  E 155m |
| **79** | Wadi El-Bada | 29° 46' 58"N  32°04' 55"E  E 162m |
| **80** | Infront of GebelEl- Ramliya | 29° 46' 33"N  32°03' 09"E  E 210m |

**Table (2): TWINSPAN output of vegetational classification showing sites number at the top and differential clusters at bottom. Species names arranged at the left-hand side and species clusters on right hand-side.**

**Sites**

1 555771127 771557 113333555557773333466 2 2 1223 1244461234474424 262466166 66

31896678905 236120489011234034571495896401 135627235027412924873880765 739689557 46

**Species Species**

**groups**

21 Deve tor -------2-----24-2-------------------------------------------------------1--------- 00000

134 Zyg dec --------------2---22221---22--2------------2-2------------------------------------ 000010

28 Arte jud ---------------------1------------------------------------------------------------ 000011

30 Broc cin -------------------2-------------------------------------------------------------- 000011

33 Echi spi --3-------------1--3-2-3--2--3334123---------------------------------------------- 000011

36 Iphi muc ------------22434-12-22-3-333-341-----------------1------------------------------- 000011

40 Laun spi ------------3-43--22--32--3-3-334----2------------1------------------------------- 000011

41 Nido aeg -------------------2-------------------------------------------------------------- 000011

50 Heli arb -------------------2-2-21--12--1-------------------------------------------------- 000011

52 Heli dig --------------------------2------------------------------------------------------- 000011

74 Ephe ala ---------------------1------------------------------------------------------------ 000011

79 Astr sie -----------------------2---------------------------------------------------------- 000011

88 Lava cor -------------------2---2-1--2--1-------------------------------------------------- 000011

89 Salv aeg ------------1--------------------------------------------------------------------- 000011

104 Call com -----------3---------------------------------------------------------------------- 000011

111 Asph ten ------------3--------------------------------------------------------------------- 000011

115 Hapl tub ------------1--2------------------------------------------------------------------ 000011

117 Scro des ------------2--------------------------------------------------------------------- 000011

32 Cent pal -----------23-------------------2-21---------------------------------------------- 000100

38 Laun nud ----------------2---------1------------2------------------------------------------ 000100

66 Gyps cap ------------4---------------------2----------------------------------------------- 000100

80 Crot aeg -------------------2-23-32-2--2-2-323---3----------------------------------------- 000100

25 Perg tom ---------------3---------------11----23------------------------------------------- 000101

27 Achi fr ---------------2------------1----1-----13----------------------------------------- 000101

35 Iflo spi --------------2-------------------2----------------------------------------------- 000101

49 Aizo can -------------------------------------1-------------------------------------------- 000101

68 Paro sin ----------------------------------1----------------------------------------------- 000101

69 Conv hys -------------------------------------2224-------------------------1--------------- 000101

91 Neur pro ----------------------------------1----------------------------------------------- 000101

109 Plat ova ----------------------------------------1----------------------------------------- 000101

120 Hyos des --------------------------------------33------------------------------------------ 000101

128 Trib mol -----------------------1----------------2----------------------------------------- 000101

101 Pani tur ------2---- 34-332---------1--2--3-2---------------------------2----1------------- 00011

119 Hyos bov -------------4-----2------------------3----2-------------------------------------- 00011

82 Reta rae --------2----2----2--------------------------------------------------------------- 00100

84 Vac t ra --2--2----1--------1---1-3---21---------1--------1--------------------- --------- -00100

116 Kick aeg ------2---- 12--------------------1----------------------------------------------- 00100

118 Lyci sha 2-43-21-----3---------2--1--2233-1------------------------1----------------------- 00100

11 Caro imb --------1-------------------------1----------------------------------------------- 001010

93 Cist tub --------2----------2-------------------------------------------------------------- 001010

1 Blep edu -1-------------------------------------------------------------------------------- 001011

4 Anab art 2--------------------------------------------------------------------------------- 001011

5 Anab set -232-44---3 1-2-------------------------------------2----------------------------- 001011

19 Halo sco -2-2------------------------------------------------------------------------------ 001011

58 Erem aeg -22------------------------------------------------------------------------------- 001011

59 Fars aeg --3244422----------2-------------------1------------------2----------------------- 001011

61 Matt l b ---------------------------------------------------------------------------------- 001011

62 Matt l l 2--------------------------------------------------------------------------------- 001011 67 Gymn dec 2---------------------------------------------------------------------------------- 001011

112 Cayl hex ------3--------------------------------------------------------------------------- 001011

72 Citr col -------2------------------------1-21--22------------3------------------------------ 00110

85 Vac t to ----------1------2-------------1---------------------------1---------------------- 00110

100 Lasi sci -------2----------------12--------2----11-------------------2-----1--------------- 00110

54 Anas hie 21----------1------11-----1------------------------1------------------------------ 00111

135 Zyg mol 2----23-----4-----------1-----11--------------------22---------------------------- 00111

53 Tric afr ---------------------2-------------------2------------323------33---------1------- 010000

6 Arth mac ------------------------------------------------------132------------------------- 010001

12 Caro ine --------------------------------------------------------3------------------------- 010001

22 Calo pro ---------------------------------------323 ------22-----------3--1-24------------- 010001

43 Puli inc ------------2---------------------------- -2--1----1------22-2-------------------- 010001

45 Reic tin ----------------------------------------------------------------2----------------- 010001

51 Heli bac ------------------------------------------ -2------------------2------------------ 010001

55 Coin tou ----------------------------------------------------------------2----------------- 010001

56 Dipl acr -----------------------------------------------------------3---------------------- 010001

71 Ipom pes -----------------------------------------------------------1---------------------- 010001

73 Cucu mel ------------------------------------------------------------1--------------------- 010001

96 Cenc div ----------------------------------------------------------------2----------------- 010001

98 Dipl fus ----------------------------------------------------------------1----------------- 010001

105 Rume cyp -----------------------------------------------------------1---------------------- 010001

114 Rese pru ---------------------------------------------------------3------------------------ 010001

127 Fors ten --------------------------------1-------3----------------4-3-----4---------------- 010001

129 Trib ter ----------------------------------------------------------------1----------------- 010001

132 Zyg bru ------1--------------------------------------------------------2------------------ 010001

20 Trag nud ------------------2----------------------1-224--1--------------------------------- 010010

29 Arte mon -------------------------------------------2-------------------------------------- 010010

39 Laun pro ---------------------------------------------2------------------------------------ 010010

46 Sene gla -------------------------------------------1-2------------------------------------ 010010

57 Dipl har --------------------------------------------22------------------------------------ 010010

64 Cleo amb ----------------------------------------2---22----------------------4------------- 010010

70 Conv lan --------------------1---------------1-------2-2-123------------------------------- 010010

86 Mon niv --------------------------------------------1------------------------------------- 010010

94 Limo pru -------------------------------------------2-------------------------------------- 010010

106 Rume spi -------------------------------------------2-------------------------------------- 010010

107 Rume ves 1------------------------------------------3--1-1----------------2---------------- 010010

31 Cent aeg -------------------2------1-----------------22------------------------------------ 010011

37 Laun muc -------------1--3----2---------------------22---22-2---1-------------------------- 010011

76 Euph ret ----------------------------------2---------2------------------------------------- 010011

7 Atri hum -------------------1--------------------------------1----------------------------- 01010

42 Pluc dio ---------------13--------------------------2---------------------2---------------- 01010

18 Halo sal ---333333233-2-------2--21-1--3-4--223--2- 22-222------323-2----2------1---------- 01011

44 Puli und -------2--3--2--1------------4-----------------2-2---3---------------------------- 01011

63 Zill spi -234---32-324-33322-22333134-4-443444-444 222---223322-4-23--33-3---3------------ 01011

121 Hyos mut ----------3-----------------------------4-------------------------33-------------- 01011

24 Lept pyr -----3----3-1-----------------3-2-2-2-32-------------3---1--2-----2-3------------- 011

113 Ochr bac --23-3-3322--2144222-23-12-4343334---1344- -2-2--21-----3---232324211----------3-- 011

130 Zyg coc 244-43434--4-33432-22--3234-4444343344333- 2--222223---44-43343424424--43--334---4 011

131 Zyg ara -------21------------2---------------------2---1-21--2-----1-------1 2----------- 100

136 Zyg Si 1-------3---------------------1---------------------------23--2-2------2---------- 100

9 Bass ind -2------------------------------------------------------4--2234-----------------24 1010

23 Cyna acu ----------------------------------2-------------1-----------3--------------------2 1010

97 Cyno dac ----------------------------------------------------------------3----------------2 1010

83 Tave aeg --------------------------------------------------------------2----1----2--------- 1011

125 Tam nil ----------- 3-3-23--2-----2--------3--2--- 1-21—21-223333333334-43-2- 44344434-44 1011

10 Bass mur ---------------------------------------- --2--------------------------------2----- 11000

17 Halo per --------------------------------------------------1----------------------2-------- 11000

92 Nitr ret -------------------2----------------------------2------3-------------- 2-22------- 11000

99 Impe cyl -------------------------------------------------2--------------------------4------11000

77 Alha gra ------------------------------------------------2-------------------34--------- 110010

2 Aerv jav --------------------------------------------------------------------------2---- 110011

16 Halop pe ------------------------------------------------------------------------2------ 110011

26 Phoe dac ---------------------------------------------------------------------2------1-- 110011

78 Astr spi --------------------------------------------------------------------1---------- 110011

87 Junu rig ---------------------------------------------------------------------4--------- 110011

102 Phra aus ------------------------------2---2-------------3---------3---------2-24443333- 110011

8 Bass eri -------------------2-------------------------------------------------------2--- 11010

65 Cleo dro -------------------2----------------------------1-------------------------2---- 11010

124 Tam aph ------------------------------4----------2------------------------------2---2-- 11010

133 Zyg alu ------------------2--------------------------------------------------------2--- 11010

126 Tam tet -----------------------------4------------2-2----------------------------2---11 11011

3 Amar vir ----------------------------------------------------------------------------2 2- 11100

81 Meli ind ----------------------------------------------------------------------------4-- 11100

103 Tric ten 2-------------------------------------------------------------------2---------- 11100

13 Chen alb ----------------------------------------------------------------------------- 2- 111010

14 Chen fic ----------------------------------------------------------------------------- 3- 111010

15 Chen mur ----------------------------------------------------------------------------- 44 111010

48 Uros pic ----------------------------------------------------------------------------- -1 111010

60 Lepi did ----------------------------------------------------------------------------- -2 111010

75 Euph pep ----------------------------------------------------------------------------- -2 111010

90 Malv par ----------------------------------------------------------------------------- 3- 111010

95 Cenc bif ----------------------------------------------------------------------------- -2 111010

108 Port ole ----------------------------------------------------------------------------- 22 111010

110 Lysi arv ----------------------------------------------------------------------------- -2 111010

122 Sola lyc ----------------------------------------------------------------------------- 3- 111010

123 Sola nig ----------------------------------------------------------------------------- 2- 111010

47 Sonc ole ------------------1----------------------2----------------------------------- 22 111011

34 Erig bon -------------------------------------3--------------------------------------- 2- 1111

**Sites**

**Clusters** 0000000000000000000000000000000000000000000000000000000000000000000011111111111

0000000000000000000000000000000000000000011111111111111111111111111100000000011

00000000000111111111111111111111111111111000000000000011111111111111000000001

0111111111100000000000000000000000111111100000000001110000000000001100111111

00000011110011111111111111111111100011110111111111 000000000011 011111

000111 000011111111111111111 000011111 0000111111 00011
